# A Label-Free Cellular Proteomics Approach to Decipher the Antifungal Action of DiMIQ, a Potent Indolo[2,3-*b*]Quinoline Agent, against *Candida albicans* Biofilms

**DOI:** 10.3390/ijms22010108

**Published:** 2020-12-24

**Authors:** Robert Zarnowski, Anna Jaromin, Agnieszka Zagórska, Eddie G. Dominguez, Katarzyna Sidoryk, Jerzy Gubernator, David R. Andes

**Affiliations:** 1 Department of Medicine, School of Medicine & Public Health, University of Wisconsin-Madison, Madison, WI 53706, USA; egdominguez@wisc.edu (E.G.D.); dra@medicine.wisc.edu (D.R.A.); 2 Department of Medical Microbiology, School of Medicine & Public Health, University of Wisconsin-Madison, Madison, WI 53706, USA; 3 Department of Lipids and Liposomes, Faculty of Biotechnology, University of Wroclaw, 50-383 Wroclaw, Poland; jerzy.gubernator@uwr.edu.pl; 4 Department of Medicinal Chemistry, Jagiellonian University Medical College, 30-688 Cracow, Poland; agnieszka.zagorska@uj.edu.pl; 5 Department of Pharmacy, Cosmetic Chemicals and Biotechnology, Team of Chemistry, Łukasiewicz Research Network-Industrial Chemistry Institute, 01-793 Warsaw, Poland; k.sidoryk@ifarm.eu

**Keywords:** *Candida*, biofilms, antifungal, proteomics, indoloquinolines, DiMIQ

## Abstract

*Candida albicans* forms extremely drug-resistant biofilms, which present a serious threat to public health globally. Biofilm-based infections are difficult to treat due to the lack of efficient antifungal therapeutics, resulting in an urgent demand for the development of novel antibiofilm strategies. In this study, the antibiofilm activity of DiMIQ (5,11-dimethyl-5*H*-indolo[2,3-*b*]quinoline) was evaluated against *C. albicans* biofilms. DiMIQ is a synthetic derivative of indoquinoline alkaloid neocryptolepine isolated from a medicinal African plant, *Cryptolepis sanguinolenta*. Antifungal activity of DiMIQ was determined using the XTT assay, followed by cell wall and extracellular matrix profiling and cellular proteomes. Here, we demonstrated that DiMIQ inhibited *C. albicans* biofilm formation and altered fungal cell walls and the extracellular matrix. Cellular proteomics revealed inhibitory action against numerous translation-involved ribosomal proteins, enzymes involved in general energy producing processes and select amino acid metabolic pathways including alanine, aspartate, glutamate, valine, leucine and isoleucine. DiMIQ also stimulated pathways of cellular oxidation, metabolism of carbohydrates, amino acids (glycine, serine, threonine, arginine, phenylalanine, tyrosine, tryptophan) and nucleic acids (aminoacyl-tRNA biosynthesis, RNA transport, nucleotide metabolism). Our findings suggest that DiMIQ inhibits *C. albicans* biofilms by arresting translation and multidirectional pathway reshaping of cellular metabolism. Overall, this agent may provide a potent alternative to treating biofilm-associated *Candida* infections.

## 1. Introduction

*Candida* spp. are opportunistic fungal pathogens capable of causing an array of infectious diseases, especially in the rapidly expanding spectrum of immunologically and medically compromised individuals [1]. *Candida albicans*, the most prevalent species of the *Candida* genus, is the most commonly isolated species, present in approximately 70% of healthy adults [2]. *C. albicans* normally lives on the skin and inside the body, in places such as the mouth, throat, gut and vagina, without causing any problems; however, the fungus is also the leading cause of invasive fungal infection in clinical settings [3]. An invasive candidiasis carries the highest, up to 50%, attributable mortality rate among all nosocomial pathogens [4]. Fungal biofilm-related infections have an enormous health impact and economic consequences, including an estimated 100,000 deaths and USD 6.5 billion in excess expenditure annually in the United States alone [5].

The occurrence of *Candida* bloodstream infection (candidemia) and invasive candidiasis has been linked to the presence of artificial medical devices, which provide an adherent surface that the pathogen colonizes and further proliferates on [6]. This process leads to the formation of biofilms, which compromise consortia of fungal cells embedded in extracellular matrix (ECM) components [7]. Biofilm formation plays a pivotal role in healthcare-associated infections and has been tightly linked to the development of resistance to available antifungal drugs [8]. An upsetting feature of biofilm-driven infections is related to the fact that fungal cells in biofilms have much higher resistance to antimicrobials as compared with their planktonic counterparts [9]. In addition, fungal cells associated with biofilm communities disperse into the bloodstream and disseminate throughout the body, too commonly leading to the patient’s death. Even though device removal is recommended for patients with *Candida*-infected medical devices, removal of these gadgets presents a serious, often deadly risk to critically ill individuals [10].

The National Institutes of Health estimate that biofilms are responsible for over 80% of all microbial infections in the United States [11]. However, there are currently no drugs that would specifically target microbial biofilms, including those formed by *C. albicans*. Lack of potent antibiofilm therapeutics makes most attempts to prevent, control or treat biofilm-based infections particularly problematic [12]. Thus, scientific research-fueled attempts to decipher mechanisms that lead to biofilm formation and continuance could result in the development of novel antibiofilm treatment strategies and specific antifungals. In fact, the scientific community has started to look for potential antibiotics to precisely target fungal biofilm cells inducting physiological switches that would yield more drug-susceptible planktonic counterparts. Although screens of multiple chemical libraries for antibiofilm activities were encouraging, it remains to be seen whether they will advance to the development of novel potent drugs [13]. A handful of promising antibiofilm agents have been identified for their efficacy, including numerous fatty acids [14], extracellular complex carbohydrates [15], multiple enzymes [16], as well as various classes of secondary metabolites, especially those of plant origin [17].

**Figure 1:**
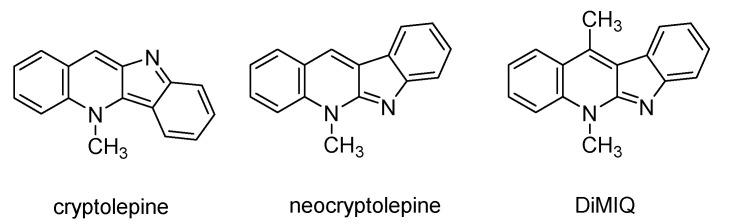
Structures of cryptolepine, neocryptolepine and DiMIQ.

Many therapeutics currently used in modern medicine are directly extracted from plants and sometimes also further chemically modified. *Cryptolepis sanguinolenta* (Lindl.) Schltr. (*Periplocaceae*) is one of the numerous medicinal African plant species which has been extensively studied in recent years [18]. This stemmed twining and scrambling shrub, which contains an orange-colored juice in the cut stem, has been used by some traditional herbalists in the treatment of broad symptoms of fever, urinary and upper respiratory tract infections, malaria, rheumatism and venereal diseases [19]. The active components found in this plant are known to be the indoquinoline alkaloids, consisting mainly of the indole and the quinoline moieties. The major alkaloid of the roots, cryptolepine (5-methyl-5*H*-indolo[3,2-*b*]quinoline, Figure 1), was reported to possess intricate biological effects, while neocryptolepine (5-methyl-5*H*-indolo[2,3-*b*]quinoline, Figure 1) is a minor alkaloid of *C. sanguinolenta* [20]. DiMIQ (5,11-dimethyl-5*H*-indolo[2,3-*b*]quinoline, Figure 1) is a synthetic analog of neocryptolepine that has been used by our research team as a base substrate for the chemical modification and synthesis of new indoquinoline derivatives [21,22]. Some of these newly synthetized amino acid and peptide conjugates showed strong preferential antifungal activities against *C. albicans* biofilms [22].

The mode of action of DiMIQ and other indolo[2,3-*b*]quinolone derivatives is rather poorly understood. These chemicals are considered DNA intercalating agents, which bind to circular double-stranded DNA and induce DNA cleavage in prokaryotic Gram-positive bacteria and eukaryotic fungi [23] as well as affecting model and natural membranes [24]. DiMIQ was also shown to stabilize the topoisomerase II-DNA cleavable complex in vitro [25]. This latter observation suggests that DiMIQ could potentially interact with other proteins and induce protein perturbations caused by drug action in treated biofilm cells. Here, we have filled this research gap by applying a label-free cellular proteomics to elucidate the specific mechanism underlying the antifungal action of DiMIQ against *C. albicans* biofilms.

## 2. Results

### 2.1. DiMIQ Is a Potent Inhibitor of C. albicans Biofilms

DiMIQ is an analogue of neocryptolepine with an additional methyl group in the 11 position. Comparison of the calculated physicochemical parameters of DiMIQ and neocryptolepine revealed that the introduction of a methyl group caused no change in acid-base properties (DiMIQ p*K*_a_ = 4.64, neocryptolepine p*K*_a_ = 4.62). The obtained value for DiMIQ is, however, smaller than that determined experimentally (7.45) [26]. Meanwhile, such modification changes the lipophilicity properties expressed as log *D* in pH 7.4. Log *D* is the decimal logarithm of distribution coefficient and describes the overall ratio of the ionized and unionized molecular forms between the polar/non-polar phases. Obtained log *D* values (DiMIQ = 4.47, neocryptolepine = 4.01) show that DiMIQ displays a greater affinity for a lipophilic environment, which may improve its penetration through biological membranes. This characteristic makes DiMIQ a promising drug candidate from a pharmacological point of view.

**Figure 2:**
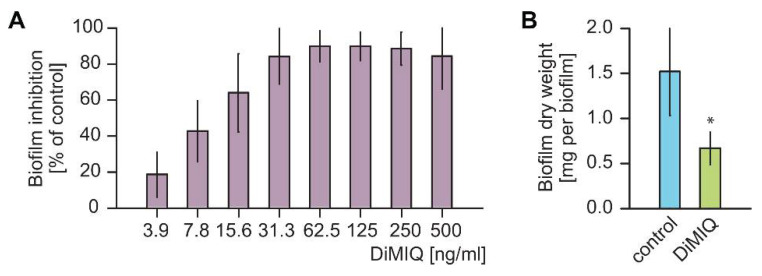
Antibiofilm action of DiMIQ against *C. albicans* biofilms. (**A**) When used at concentrations greater than 31.3 ng/mL, DiMIQ completely inhibited fungal biofilm formation in vitro. The drug was applied after 6 and 24 h of biofilm growth and its antibiofilm impact was determined using an XTT reduction assay. Data are expressed as percent reduction compared to untreated controls. Mean values shown. (**B**) DiMIQ applied at the ED_50_ concentration of 10.8 ng/mL dramatically reduced biofilm dry weight yield. Mean ± SD values shown. * *p* < 0.01.

Our initial experiments were designed to evaluate the antifungal activity of DiMIQ against *C. albicans* biofilms. We utilized a 96-well plate assay platform for the assessment of the metabolic activity of fungal biofilms using an XTT (2,3-bis[2-methoxy-4-nitro-5-sulfophenyl]-2*H*-tetrazolium-5-carboxanilide inner salt) endpoint. DiMIQ was tested in the concentration range up to 500 ng/mL and applied twice, at 6 and 24 h biofilm growth time points (Figure 2A). Doses of DiMIQ of 31.3 ng/mL and above completely inhibited the metabolic activity of *C. albicans* biofilms. We further calculated the drug concentration associated with a 50% reduction in the metabolic activity of the treated fungal biofilms (ED_50_), which was observed at 10.8 ± 0.2 ng/mL. All subsequent experiments on *C. albicans* biofilms were carried at the ED_50_ concentration. When used during biofilm seeding in a 6-well plate assay, this selected dose of DiMIQ had a tremendous effect upon biofilm formation yielding only approximately 44 ± 7% of dry biomass as compared to untreated controls (Figure 2B).

### 2.2. DiMIQ Alters C. albicans Biofilm Cell Walls

**Figure 3:**
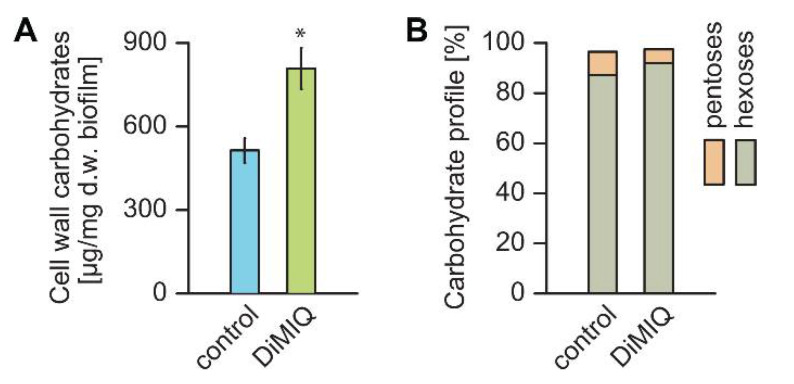
DiMIQ-induced alterations in cell walls of *C. albicans* biofilms. (**A**) Exposure of biofilm fungal cells to DiMIQ resulted in increased cell wall production. Mean ± SD values shown. * *p* < 0.01. (**B**) DiMIQ-treatment affected percentage carbohydrate profiles of fungal cell walls by increasing the content of hexoses and simultaneously reducing pentoses. Mean values are shown.

The biochemical composition of cell walls is relatively conserved among fungi and crucial for cell wall integrity, which further determines accommodative stress response to both abiotic and biotic factors. We hypothesized that the exposure of *C. albicans* biofilms to DiMIQ may lead to alterations in overall cell wall production and composition. Compared to untreated controls, the fraction of fungal cell walls isolated from DiMIQ-treated *C. albicans* biofilms was significantly increased by nearly 57% (Figure 3A). The composition of these cell walls was also altered in comparison to untreated controls. Hexoses, such as glucose and mannose, build the major cell wall carbohydrate polymers, glucans and mannans. Compared to untreated fungal biofilms, the level of hexoses in the cell walls of DiMIQ-treated *C. albicans* biofilms was elevated by 4.8% to a total of 92.2%. In addition, the exposure of fungal biofilms to DiMIQ had a minute detrimental effect on the content of cell wall pentoses (arabinose, ribose, xylose), which was reduced from 9.2% in untreated controls down to 5.2% (Figure 3B).

### 2.3. DiMIQ Affects the ECM of C. albicans Biofilm

**Figure 4:**
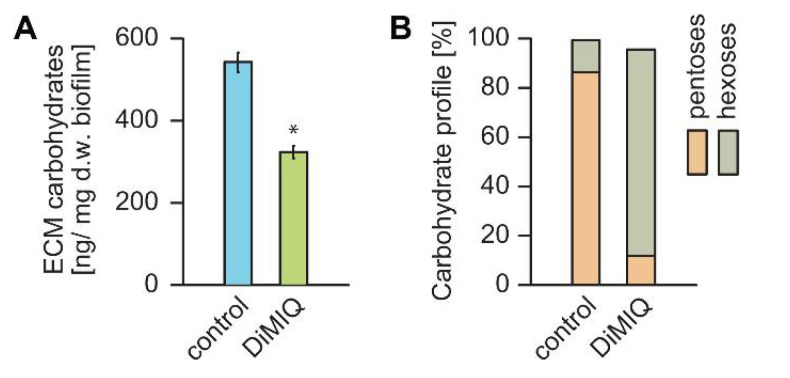
Negative impact of DiMIQ on the *C. albicans* biofilm extracellular matrix (ECM). (**A**) The presence of DiMIQ dramatically reduced the overall amount of ECM in fungal biofilms by nearly 40%. Mean ± SD values shown. * *p* < 0.01. (**B**) DiMIQ treatment massively modulated the percentage carbohydrate profile of the ECM by a dramatic reduction in pentoses and a simultaneous increase in hexoses. Mean values shown.

A key feature of fungal biofilms is their ability to produce the extracellular matrix (ECM), which embeds cells and constitutes an external protective barrier to the surrounding environment. Since the ECM is considered a crucial factor in biofilm resistance to antifungals, we hypothesized that DiMIQ may have a strong effect on both the content and the quality of the *C. albicans* biofilm ECM. Compared to untreated controls, the ECM was reduced by nearly 40% in fungal biofilms grown in the presence of DiMIQ (Figure 4A). The ECM composition in control biofilms consisted of 86.3% pentoses and 13% hexoses. The content of arabinose, the main ECM monocarbohydrate, in untreated controls was 79.7%. The ECM profile was strongly affected in the presence of DiMIQ, which noticeably reduced the level of pentoses down to 11.9% with arabinose content decreased to 5.8%. These negative changes were accompanied by an increase in hexoses, with glucose content reaching 73.3% (Figure 4B).

### 2.4. DiMIQ Induces Changes in the Cellular Proteome of C. albicans Biofilm Cells

**Figure 5:**
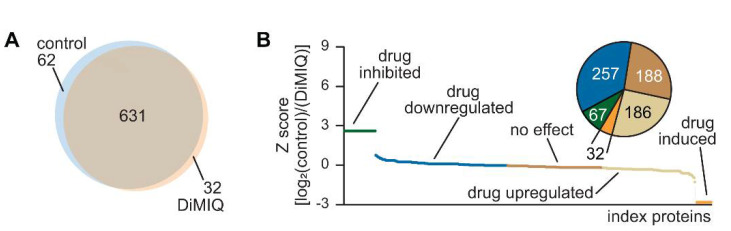
DiMIQ-induced changes in the cellular profile of *C. albicans* biofilms. (**A**) A set of 725 proteins were identified in the tested fungal biofilms. The control proteome consisted of 693 proteins, whereas the DiMIQ-exposed proteome consisted of 663 proteins. The pool of common proteins contained 631 proteins. (**B**) Only 188 proteins were not affected by the DiMIQ treatment in the cellular proteome of *C. albicans* biofilms. There was a pool of 218 proteins that were either drug-upregulated or induced, whereas 319 proteins were either downregulated or completely inhibited by the drug.

A total of 725 proteins were identified from *C. albicans* biofilm cells (Figure 5A). The proteome of untreated control biofilm cells consisted of 693 proteins, whereas 663 proteins were found in the proteome of DiMIQ-treated biofilm cells. The tested indoquinoline derivatives either completely inhibited the synthesis of 62 protein or decreased the production of 257 proteins. DiMIQ also upregulated or induced the production of 186 and 32 proteins, respectively. DiMIQ had no effect on the production of 188 proteins (Figure 5B). Calculated Z-score values are provided in Table S1.

DiMIQ and other indoquinoline alkaloids are considered DNA intercalating agents capable of interacting with DNA topoisomerase II, an enzyme involved in chromosome-associated DNA replication [23]. Our proteomics analysis identified this specific enzyme (TOP2, CAALFM_C406600WA), which was 2.4-fold upregulated in the presence of the tested drug (Table S1).

**Figure 6:**
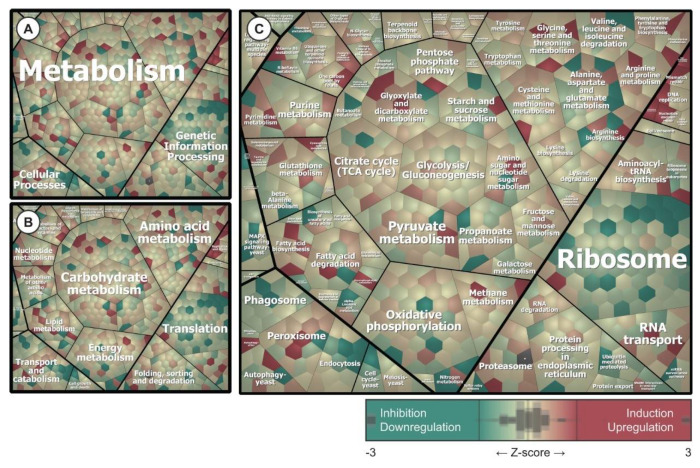
Functional mapping of the cellular proteome revealed targets for DiMIQ in *C. albicans* biofilms. (**A**–**C**) Proteins were grouped based on assignments of Kyoto Encyclopedia of Genes and Genomes (KEGG) functional categories. Voronoi treemap layouts were used to visualize identified proteins, which were arranged into smaller clusters inside higher level regions. This hierarchical classification scheme was mapped to a color ramp starting with green (inhibited and downregulated proteins), passing yellow (no significant drug effect) and reaching red (upregulated and induced proteins). Quantitative differences in the proteome are based on calculated Z-score values (Table S1).

The identified proteins were functionally mapped based on pathway assignments in the UniProt and Kyoto Encyclopedia of Genes and Genomes (KEGG) databases (Figure 6) [16,27,28,29]. A total of 394 different functions were represented by 91 enzymes classified in the pool of DiMIQ-upregulated/induced proteins. The majority of proteins classified into this pool were involved in carbon metabolism (31 proteins), biosynthesis of amino acids (22 proteins), biosynthesis of cofactors (15 proteins), oxidative phosphorylation (14 proteins), glycolysis/gluconeogenesis (13), citrate cycle (TCA cycle) (13 proteins), pyruvate metabolism (12 proteins), glyoxylate and dicarboxylate metabolism (11 proteins). Other, less abundant functional assignments involved the following pathways: methane metabolism (9 proteins), peroxisome (9 proteins), 2-oxycarboxylic acid metabolism (9 proteins). DiMIQ induced enzymes that were involved in amino acid metabolism such as cysteine and methionine metabolism (8 proteins), arginine and proline metabolism (7 proteins), glycine, serine and threonine metabolism (6 proteins), arginine biosynthesis (5 proteins), phenylalanine, tyrosine and tryptophan biosynthesis (4 proteins). In addition, smaller numbers of enzymes were mapped to pathways related to the metabolism of carbohydrates (fructose and mannose metabolism, starch and sucrose metabolism, galactose metabolism) and nucleic acids (aminoacyl-tRNA biosynthesis, RNA transport, purine metabolism, pyrimidine metabolism, RNA degradation, amino sugar and nucleotide sugar metabolism).

There were 378 distinct functions assigned to 74 enzymes in the pool of DiMIQ downregulated or inhibited proteins in *C. albicans* biofilms. The majority of proteins in this group were classified into the following clusters: ribosome (59 proteins), oxidative phosphorylation (16 proteins), carbon metabolism (14 proteins), phagosome (10 proteins), protein processing in endoplasmic reticulum (10 proteins), pyruvate metabolism (8 proteins), biosynthesis of cofactors (8 proteins). DiMIQ inhibited certain aspects of amino acid metabolism such as the biosynthesis of amino acids (11 proteins), alanine, aspartate and glutamate metabolism (10 proteins), valine, leucine and isoleucine degradation (6 proteins) and beta-alanine metabolism (6 proteins). DiMIQ also negatively impacted pathways involved in RNA transport (9 proteins), purine metabolism (6 proteins) or starch and sucrose metabolism (7 proteins).

## 3. Discussion

Biofilms constitute an ideal and unique lifestyle niche for microorganisms that provide suitable environmental conditions for growth and proliferation, even in the presence of toxic antifungals. In fact, microbial cells living in biofilms are significantly more resistant to antimicrobial agents than their planktonic counterparts [30,31]. As biofilms strongly contribute to the fungal pathogenicity, unraveling and understanding the mechanisms of drug resistance is a key approach to develop antibiofilm treatment regimens and successfully deal with the problem of medical device-associated fungal infections [32]. Because of the lack of effective antifungal therapy against biofilm infections, the only recommended treatment for *Candida* biofilm infections is removal of the infected device [33]. Over the last few decades, only a handful of novel approaches that theoretically prevent and control biofilm formation and development have been developed. These antibiofilm strategies involve non-adhesive coatings and cell repellents applied to medical devices, coatings that actively release incorporated antimicrobials and biofilm inhibitors and coatings with covalently tethered antimicrobials [34,35]. Recent years have also brought the emergence of resistance to the most common antifungal drugs; thus, there is an urgent need for the development of alternative antifungal agents.

The indoquinoline alkaloids of *C. sanguinolenta* constitute a group of promising natural metabolites with an impressive plethora of biological activities. Some of these chemicals, such as DiMIQ, have been used as substrates for the chemical modification and synthesis of new indoquinoline derivatives with strong preferential antifungal activities against *C. albicans* biofilms [22]. The mechanistic aspect of indoquinoline derivatives’ action remains rather poorly understood. Early studies implied that these chemicals may interact with DNA as well as target DNA topoisomerase II [23]. As DiMIQ and its derivatives appear as promising antibiofilm agents against *C. albicans* biofilms, defining the targeted pathways and deciphering the mechanisms of action of these compounds would be of great value for the design of innovative drug therapies in the future.

In this study, we showed DiMIQ as an effective and potent inhibitor of *C. albicans* biofilm formation. At the ED_50_ concentration of 10.8 ng/mL, the drug significantly reduced both metabolic activity and dry weight biomass (Figure 3). The tested indoquinoline derivative had a strong effect on the quantity of biofilm cell walls and resulted in nearly a 60% increase in cell wall dry weight biomass as compared to untreated controls. It is worth mentioning that DiMIQ at the concentrations tested in this study was not toxic to mammalian cells [36]. In addition, DiMIQ affected the cell wall composition profile, which led to elevated levels of glucans and mannans and simultaneous reductions in cell wall pentoses. This finding suggests that the antibiofilm action of DiMIQ overwhelms *C. albicans* biofilms, which in response produce excessive amounts of cell walls. These structures are crucial for fungal cells as they represent the multifactorial interface for interactions between the cell interior and the outer environment and play an important role in drug resistance [37].

Like fungal cell walls, the ECM produced by *C. albicans* biofilms provides a protective barrier to antifungal drugs by drug sequestration and limiting biofilm penetration [7,31]. In this study, we showed that DiMIQ inhibited ECM production by almost 40% (Figure 4). The drug had a strong effect on the chemical composition of the biofilm ECM, which, in the presence of DiMIQ, primarily consisted of glucose and mannose and small amounts of arabinose. The latter is considered an extracellular carbon nutrient reservoir that is utilized by biofilm fungal cells under nutritionally less favorable conditions, such as starvation or exposure to antifungals (Zarnowski and Andes, unpublished). This finding also indicates that the antifungal activity of DiMIQ targets certain aspects of the metabolism in *C. albicans* biofilm cells.

In this study, we applied a label-free proteomics analysis to explain the DiMIQ-induced protein abundance changes observed in *C. albicans* biofilms and elucidate the drug’s mode of action. We discovered that DiMIQ induced notable alterations in the cellular proteomic profile (Figure 5). A total of 725 unique proteins were identified in the tested fungal biofilms. DiMIQ had a negative effect on 319 identified proteins that were either partially downregulated or completely inhibited. Interestingly, DNA topoisomerase II (TOP2) appeared to be not a direct target for DiMIQ, but its action on this enzyme seemed more indirect. Apparently, DiMIQ interacted with the DNA structure, but more direct interactions were determined with nucleosome core proteins (HHT21, HHF1, HTA2, HTZ1 and HTB1). It is therefore quite likely that the drug intercalated fragments of fungal DNA, which is a substrate for TOP2. As the DNA substrate was inactivated in the presence of DiMIQ, TOP2 was produced by *C. albicans* cells at elevated levels, probably via a yet-to-be-identified feedback loop mechanism.

**Figure 7:**
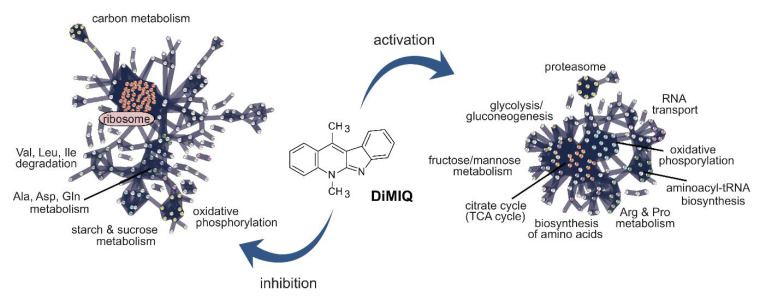
Visualization of major DiMIQ-induced alterations in metabolic pathway networks in the *C. albicans* biofilm cellular proteome. Interactions between proteins were predicted using the Search Tool for the Retrieval of Interacting Genes/Proteins (STRING).

DiMIQ, the tested indoquinoline derivative, appeared as a potent inhibitor of protein synthesis (Figure 6) and its action had an enormous impact on other metabolic pathways (Figure 7). The drug had a strong inhibitory effect on numerous ribosomal proteins (RPL37B, RPL2, RPL82, RPS17B, C4YDX8, RPS14B, RPL6, RPS16A, RPL13, RPL10, C4YF39, RPS21, RPS21B, RPL16A, RPL4B, RPL14, RPL27A, RPL20B, RPS6A, RPL39, RPL17B, C4YIL5, C4YIP1, RPL21A, RPS9B, RPL11, C4YK99, RPL3, RPL28, RPS3, RPS27, RPS20, C4YMQ1, RPL18, RPS19A, RPS0, RPS7A, RPL12, RPL9B, RPL19A, RPL35, C4YR43, RPS5, RPS25B, RPL10A, RPL23A, RPS28B, RPS18, RPL5 and RP10). Interestingly, several of these targeted proteins represented the protein SH3-like domain superfamily (RPL2, RPL6, C4YF39, RPL14, RPL27A and RPL21A), which is involved in the translation process. We also identified a pool of GTP-binding proteins that were downregulated by the tested agent (TEF1, RAC1, SEP7, RAS1, RSR1, CDC10, ARF3, CDC11, ADE12 and GSP1).

DiMIQ inhibited several important cellular metabolism pathways related to cellular energy production, such as oxidative phosphorylation (orf19.6035, VMA8, VPH1, orf19.364, QCR8, CYT1, ATP14, orf19.1480, VMA5, VMA4, VMA13, SDH2, orf19.7590, PMA1, orf19.1682 and NUC2) and pyruvate metabolism (orf19.6066, ERG10, LYS22, LEU42, MDH1-3, FUM11, GLX3 and orf19.5611) (Figure 6 and Figure 7). Changes in the pyruvate metabolism pathway usually affect other metabolic traits, such as alanine, aspartate and glutamate metabolism. In fact, our study revealed that DiMIQ negatively impacted several proteins in this pathway (GAD1, GLT1, GDH3, UGA1, GLN1, ADE13, ARG1, CPA2, GFA1 and ADE12). The drug impacted the propanoate metabolism pathway (EHD3, ALD6, UGA1, ERG10 and orf19.5611), which directly intersects with the valine, leucine and isoleucine degradation pathway (EHD3, orf19.6066, ALD6, UGA1, ERG10 and POT1). We also detected some derogatory action of DiMIQ on enzymes involved in beta-alanine metabolism (GAD1, EHD3, orf19.6066, ALD6 and UGA1). The activity of this route intersects with the downregulated alanine, aspartate and glutamate metabolic pathway. In humans, glutamine is the most abundant circulating non-essential amino acid and is central for whole-body nitrogen balance as well as for the nitrogen balance of each organ; however, glutamate plays essential roles in most biological growth and biofilm formation [38]. In fact, there are actively formed glutamine gradients in microbial biofilms, which determine co-dependence between peripheral and central cells for glutamine synthesis, which gives rise to metabolic commensalism [39]. Based on our findings presented in this study, we conclude that DiMIQ acts as an effective inhibitor of ribosomes and subsequent translation, as well as altering processes related to cellular energy production along with corresponding amino acid metabolic pathways. We also discovered that DiMIQ inhibited glutamine metabolic pathways, which may potentially lead to the identification of vital targets and promote the development of novel antibiofilm drugs in the future [38,40].

Our experimental results showed that some DiMIQ-counteracting metabolic efforts occurred in the proteome of *C. albicans* biofilms (Figure 6 and Figure 7). The drug either upregulated or induced a total of 218 proteins. Most of those proteins were involved in the following pathways: citrate cycle (TCA cycle) (IDH1, LSC1, PDA1, SDH12, PDB1, MDH1-1, IDP1, IDH2, CIT1, LSC2, LPD1, PCK1, MDH1 and KGD1), glycolysis/gluconeogenesis (ENO1, ADH2, ACS2, PDA1, PDB1, FBA1, CDC19, GPM1, ACS1, HXK2, LPD1, FDH3, PCK1 and orf19.6178), oxidative phosphorylation (COX5, orf19.4758, ATP7, SDH12, C4YI42, ATP2, ATP4, COX13, COX4, ATP16, ATP3, ATP5 and ALI1), pyruvate metabolism (MLS1, ACS2, PDA1, PDB1, MDH1-1, CDC19, ACS1, LPD1, PCK1, MDH1, ACC1 and ACH1), glyoxylate and dicarboxylate metabolism (MLS1, GCV2, ICL1, MDH1-1, CIT1, FDH1, LPD1, MDH1, SHM1), 2-oxocarboxylic acid metabolism (IDH1, AAT1, IDP1, ARO8, IDH2, CIT1, BAT22 and ALT1). DiMIQ also upregulated peroxisomal enzymes (orf19.3684, SOD2, CAT2, IDP1, FAA4, FOX3, CTN3 and FAA2-3). Some of these pathways were also inhibited by the drug, which indicates the existence of multiple functionally redundant metabolic routes and underlies a robust metabolism-adaptable biofilm survival strategy [41]. Overall, stimulation of these metabolic processes leads to increased energy production, which is probably quite limited in fungal biofilm cells in the presence of DiMIQ. In addition, these pathways are functionally linked to other amino acid-based metabolic routes such as arginine and proline metabolism (PRO3, CAR2, AAT1, SPE3, PRO2, CAR1 and PUT2), cysteine and methionine metabolism (MDH1-1, AAT1, ARO8, SPE3, MET6, MDH1 and BAT22), arginine biosynthesis (AAT1, GDH2, ALT1 and CAR1). DiMIQ also upregulated the production of proteins involved in aminoacyl-tRNA biosynthesis (TYS1, VAS1, FRS1, SES1, ILS1, orf19.6701 and GUS1). Aminoacyl-tRNAs are recognized as potential antimicrobial drug targets and their inhibition has proven to be an effective antimicrobial strategy that impedes an essential step of protein synthesis [42]. Our results showed that DiMIQ did inhibit ribosomal proteins and stimulated the biosynthesis of aminoacyl-tRNA molecules. Thus, our finding may be explained by the presence of specific, yet unidentified metabolic feedback loops resulting from the indirect inhibitory action of DiMIQ on ribosomal proteins.

We also discovered that DiMIQ upregulated sets of proteins involved in carbohydrate metabolism, such as fructose and mannose metabolism (FBA1, PMI1, HXK2, orf19.6178 and MPG1) or pentose phosphate pathway (TKL1, FBA1, TAL1, orf19.6178 and RKI1). This phenomenon, along with the stimulation of fatty acid metabolism, could justify the observed quantitative and qualitative alterations in *C. albicans* biofilm fungal cell walls.

In conclusion, we demonstrate that DiMIQ exhibits excellent antifungal activity against *C. albicans* biofilms. The drug exerted a robust antibiofilm action, which altered biofilm formation and growth, cell walls, the ECM and multiple metabolic pathways. These findings indicate that DiMIQ and DiMIQ-based derivatives could provide an alternative approach to treating biofilm-associated *Candida* infections in the future.

## 4. Materials and Methods

### 4.1. Microbes and Chemicals

DiMIQ was synthesized according to the method described previously [43]. The negative logarithm of the acid dissociation constant (p*K*_a_) and the decimal logarithm distribution coefficient (log *D*) were calculated using the MarvinSketch software (version 19.26.0, ChemAxon Ltd., Budapest, Hungary). All biofilm experiments were done on *C. albicans* SN250 strain (SC5314 background) [44], which was stored in 15% glycerol frozen at −80 °C. The fungus was routinely maintained on yeast extract peptone (YPD) agar plates (1% yeast extract, 2% Bacto™ peptone, 2% dextrose, 2% Bacto™ agar), whereas liquid cultures were grown in broth YPD (1% yeast extract, 2% Bacto™ peptone, 2% dextrose) rotating at 200 rpm at 30 °C. All media components were manufactured by Becton, Dickinson and Company (Franklin Lakes, NJ, USA). For biofilm assays, strains were cultured in filter sterilized Roswell Park Memorial Institute (Buffalo, NY, USA) medium 1640 (RPMI), buffered with 4-morpholinepropanesulfonic acid (MOPS) (Thermo Fisher Scientific, Waltham, MA, USA) and pH adjusted to 7.0 [45]. All reagents used were acquired through the UW-Madison Material Distribution Services (Madison, WI, USA).

### 4.2. Evaluation of Metabolic Activity in Fungal Biofilms

Ninety-six-well flat-bottom polystyrene plates were used to assess biofilm susceptibility to drug treatment. Fungal cell inocula (10^6^ cells/mL) were prepared out of overnight yeast cultures in YPD at 30 °C, followed by dilution in RPMI-MOPS based on count numbers with an automated Countess™ II cell counter (Invitrogen, Thermo Fisher Scientific, Waltham, MA, USA). One hundred μL of yeast cells per well was seeded and inoculated plates were incubated at 37 °C. After a 6-h biofilm formation period in the wells of 96-well microtiter plates, the biofilms were washed twice with phosphate-buffered saline (PBS, pH 7.2) in order to remove nonadherent cells, followed by the addition of DiMIQ and fresh RPMI medium. The drug was used at concentrations up to 500 ng/mL. The procedure, including the drug treatment, was repeated after 24 h and the plates were incubated for an additional period of 24 h, followed by the XTT assay [46]. Briefly, solutions of XTT (2,3-bis[2-methoxy-4-nitro-5-sulfophenyl]-2*H*-tetrazolium-5-carboxanilide inner salt; 0.75 mg/mL) and PMS (5-methylphenazinium methyl sulfate; 3.20 mg/mL) were prepared fresh for each set of assays and were kept away from light. Both reagants were purchased from Thermo Fisher Scientific (Waltham, MA, USA). To each well, 90 μL XTT and 10 μL phenazine methosulfate were added and incubated in the dark at 37 °C for 1 h. Absorbances at 492 nm were measured using an automated Cytation 5 imaging reader (BioTek, Winooski, VT, USA). The percent reduction in biofilm growth was calculated using the reduction in absorbance compared to that of controls with no antifungal treatment.

### 4.3. C. albicans Biofilm Cell Wall and ECM Analyses

Six-well polystyrene plates were used to grow fungal biofilms for cell wall isolation and subsequent analysis. Fungal cell inocula were prepared as described above and biofilms were seeded with 10^6^ yeast cells per well. The nonadherent cells were removed after a 60-min-long adherence incubation and 1 mL of fresh RPMI medium was applied to each well. The biofilms were grown on an orbital shaker set at 50 rpm at 37 °C for 24 h; then, the medium was replaced with fresh RPMI or RPMI supplemented with DiMIQ followed by the incubation for another 24 h. Biofilms were removed from wells with a sterile spatula and harvested in sterile water (1 mL/well). The aliquots were combined in a 15-mL Falcon tube and the biofilm biomass was subjected to sonication in a water bath sonicator for 20 min. The sample was next centrifuged at 2880× *g* at 4 °C for 20 min. This step separated the dissolved ECM from ECM-free fungal cells. Five-ml of the collected filter-sterilized ECM suspension was placed in a clear 8-mL glass screw thread vial, dried overnight at 60 °C and subjected to monocarbohydrate analysis [7,8]. Pelleted biofilm fungal cells were washed twice with PBS, resuspended in PBS and broken with glass beads five times for 1 min each [37]. Cell walls were harvested by centrifugation, washed 5 times with water, dried overnight at 60 °C and dry weights were determined. The isolated cell walls were subjected to monocarbohydrate analysis [7,8].

Carbohydrates in the biofilm ECM and cell wall fractions were analyzed based on the modified procedures reported elsewhere [7]. Monosugars were converted to alditol acetate derivatives [47] and then identified and quantified by gas chromatography on a Shimadzu GC-2010 system (Shimadzu, Columbia, MD, USA). A Crossbond 50% cyanopropylmethyl/50% phenylmethyl polysiloxane column was used (15 m × 0.25 mm with 0.25 μm film thickness, RTX-225, Restek, Bellefonte, PA, USA). The gas chromatography conditions were as follows: injector at 220 °C, FID detector at 240 °C, and a temperature program of 215 °C for 2 min, then 4 °C/min up to 230 °C before holding for 11.25 min, run at constant linear velocity of 33.4 cm/s and split ratio of 50:1.

### 4.4. C. albicans Biofilm Proteomics

Enzymatic “in liquid” digestion and mass spectrometric analysis was done at the Mass Spectrometry Facility, Biotechnology Center, University of Wisconsin—Madison. Two hundred μg of protein was extracted by precipitation with 15% TCA/60% acetone and then incubated at −20 °C for 30 min. The matrix or vesicle preparation was centrifuged at 16,000× *g* for 10 min, and the resulting pellets were washed twice with ice-cold acetone, followed by an ice-cold MeOH wash. Pelleted proteins were resolubilized and denatured in 10 μL of 8 M urea in 100 mM NH_4_HCO_3_ for 10 min, then diluted to 60 μL for tryptic digestion with the following reagents: 3 μL of 25 mM DTT, 4.5 μL of acetonitrile, 36.2 μL of 25 mM NH_4_HCO_3_, 0.3 μL of 1M Tris-HCl, and 6 μL of 100 ng/μL Trypsin Gold solution in 25 mM NH_4_HCO_3_ (Promega, Madison, WI, USA). Digestion was conducted in two stages: first, overnight at 37 °C; then, an additional 4 μL of trypsin solution was added and the mixture was incubated at 42 °C for an additional 2 h. The reaction was terminated by acidification with 2.5% TFA to a final concentration of 0.3% and then centrifuged at 16,000× *g* for 10 min. Trypsin-generated peptides were analyzed by nanoLC-MS/MS using the Agilent 1100 nanoflow system (Agilent, Santa Clara, CA, USA) connected to a hybrid linear ion trap-orbitrap mass spectrometer (LTQ-Orbitrap, Thermo Fisher Scientific, Waltham, MA, USA) equipped with a nanoelectrospray ion source. Capillary HPLC was performed using an in-house fabricated column with an integrated electrospray emitter, as described elsewhere [48]. Sample loading and desalting were achieved using a trapping column in line with the autosampler (Zorbax 300SB-C18, 5 μm, 5 × 0.3 mm, Agilent, Santa Clara, CA, USA). The LTQ-Orbitrap was set to acquire MS/MS spectra in a data-dependent mode as follows: MS survey scans from 300 to 2000 *m*/*z* were collected in profile mode with a resolving power of 100,000. MS/MS spectra were collected on the five most abundant signals in each survey scan. Dynamic exclusion was employed to increase the dynamic range and maximize peptide identifications. Raw MS/MS data were searched against a concatenated *C. albicans* amino acid sequence database using an in-house MASCOT search engine [49]. Identified proteins were further annotated and filtered to 1.5% peptide and 0.1% protein false discovery rate with Scaffold Q+ version 4.10.0 (Proteome Software Inc., Portland, OR, USA) using the protein prophet algorithm [50].

### 4.5. Functional Mapping of the C. albicans Biofilm Proteomes

The *C. albicans* control and DiMIQ-treated biofilm proteomes were analyzed using the UniProt Knowledgebase data, which contain reviewed UniProtKB/Swiss-Prot entries [29,51]. The data were next analyzed using the Kyoto Encyclopedia of Genes and Genomes (KEGG) [27,28]. In addition, the data were clustered using STRING (the Search Tool for the Retrieval of Interacting Genes/Proteins) v.11.0 [52]. Each protein predicted from the *C. albicans* genome assigned a KEGG Ontology ID (KOID) was obtained, and the specific pathway and superpathway membership information retained. This was then correlated with the experimental proteome data, and the number of proteins expressed within a given pathway was then determined. Tabulated proteins were presented as a percentage out of the total number of proteins predicted to belong to a given pathway from the *C. albicans* genome, as determined by KEGG. The visualization of relative quantities of biofilm proteins was also done using KEGG protein functional categorization. On the basis of this hierarchical classification scheme, Voronoi treemaps were constructed [53]. This approach divides screen space according to hierarchy levels in which the main functional categories determine screen sections on the first level, subsidiary categories on the second level, and so forth. The polygonic cells of the deepest level represented functionally classified proteins and were colored according to the relative abundance of each protein that was determined based on total counts of corresponding trypsin-digested peptides. Voronoi treemaps were prepared with Paver (version 2.1.9, DECODON Software UG, Greifswald, Germany).

### 4.6. Statistical Analysis

Datasets were analyzed using one-way analysis of variance (ANOVA) and the post-hoc Tukey Honestly Significance Difference test and with Bonferroni and Holm interference comparisons. Statistically significant outliers were identified based on the Grubbs’ test. Data were processed with GraphPad Prism 9 for Windows 64-bit (version 9.0.0).

## Data Availability

The data presented in this study are available in the provided Supplementary material (Table S1).

## Supplementary Materials

Supplementary materials can be found at https://www.mdpi.com/1422-0067/22/1/108/s1.

## Abbreviations

DiMIQ	5,11-dimethyl-5*H*-indolo[2,3-*b*]quinoline
ECM	Extracellular matrix
KEGG	Kyoto Encyclopedia of Genes and Genomes
STRING	Search Tool for the Retrieval of Interacting Genes/Proteins
XTT	2,3-bis[2-methoxy-4-nitro-5-sulfophenyl]-2*H*-tetrazolium-5-carboxanilide inner salt
