# Supplementary material for: A Label-Free Cellular Proteomics Approach to Decipher the Antifungal Action of DiMIQ, a Potent Indolo[2,3-b]Quinoline Agent, against Candida albicans Biofilms
Source: Int J Mol Sci. 2020 Dec 24;22(1):108. doi: 10.3390/ijms22010108 (PMC7795236; doi:10.3390/ijms22010108)
Supplement: Supplementary file 1 [file ijms-22-00108-s001.pdf]

A Label-Free Cellular Proteomics Approach to Decipher the Antifungal Action of DiMIQ, a Potent Indolo[2,3-*b*]Quinoline Agent, against *Candida albicans* Biofilms

Robert Zarnowski [1,2*], Anna Jaromin [3*], Agnieszka Zagórska [4], Eddie G. Dominguez [1,2], Katarzyna Sidoryk [5], Jerzy Gubernator [3] and David R. Andes [1,2]

1 Department of Medicine, School of Medicine & Public Health, University of Wisconsin-Madison, Madison, WI 53706, USA; egdominguez@wisc.edu (E.G.D.); dra@medicine.wisc.edu (D.R.A.)
2 Department of Medical Microbiology, School of Medicine & Public Health, University of Wisconsin-Madison, Madison, WI 53706, USA
3 Department of Lipids and Liposomes, Faculty of Biotechnology, University of Wroclaw, 50-383 Wroclaw, Poland; jerzy.gubernator@uwr.edu.pl
4 Department of Medicinal Chemistry, Jagiellonian University Medical College, 30-688 Cracow, Poland; agnieszka.zagorska@uj.edu.pl
5 Department of Pharmacy, Cosmetic Chemicals and Biotechnology, Team of Chemistry, Łukasiewicz Research Network-Industrial Chemistry Institute, 01-793 Warsaw, Poland; k.sidoryk@ifarm.eu
* Correspondence: rzarnowski@medicine.wisc.edu (R.Z.); anna.jaromin@uwr.edu.pl (A.J.); Tel.: +1-608-265-8578 (R.Z.); +48-71-3756203 (A.J.)

Label-Free Cellular Proteomics of *Candida albicans* biofilms treated with DiMIQ

Identified Proteins	Accession #	Alternate ID	Gene names (ORF)	WT	DIMIQ	Z SCORE
**Proteins induced by DiMIQ**						
Arginase (EC 3.5.3.1)	A0A1D8PP00	CAR1	CAALFM_C504490CA	0.000	6.648	drug induced
Glucan 1,3-beta-glucosidase BGL2 (EC 3.2.1.58) (Exo-1	Q5AMT2	BGL2	CAALFM_C402250CA	0.000	4.432	drug induced
GPI-anchored hemophore RBT5 (Repressed by TUP1	Q59UT4	RBT5	CAALFM_C400130WA	0.000	4.432	drug induced
Uncharacterized protein	Q59RB7	orf19.6845	CAALFM_C104510WA	0.000	4.432	drug induced
Phenylpyruvate decarboxylase	Q59MU3	ARO10	CAALFM_CR06860CA	0.000	3.324	drug induced
Uncharacterized protein	A0A1D8PPB2	orf19.1179	CAALFM_C600200CA	0.000	3.324	drug induced
SAP domain-containing protein	A0A1D8PIF1	orf19.200	CAALFM_C209040WA	0.000	3.324	drug induced
Serine hydroxymethyltransferase (EC 2.1.2.1)	A0A1D8PRB3	SHM1	CAALFM_C703330CA	0.000	2.216	drug induced
Glycerol-3-phosphate dehydrogenase (EC 1.1.5.3)	A0A1D8PMP1	GUT2	CAALFM_C406760WA	0.000	3.324	drug induced
MRX complex DNA-binding subunit	A0A1D8PJD1	RAD50	CAALFM_C302000WA	0.000	3.324	drug induced
TORC2 complex subunit	A0A1D8PTJ4	TSC11	CAALFM_CR07580CA	0.000	3.324	drug induced
Proteasome endopeptidase complex (EC 3.4.25.1)	Q59Z65	PRE2	CAALFM_C206820CA	0.000	2.216	drug induced
Pentafunctional AROM polypeptide [Includes: 3-dehy	Q5AME2	ARO1	CAALFM_C400890WA	0.000	2.216	drug induced
Eukaryotic translation initiation factor 3 subunit A (eI	Q59PL9	RPG1A	CAALFM_C112770WA	0.000	2.216	drug induced
Glycine cleavage system P protein (EC 1.4.4.2)	A0A1D8PE97	GCV2	CAALFM_C108400CA	0.000	2.216	drug induced
Phosphoserine aminotransferase (EC 2.6.1.52)	Q59P52	SER1	CAALFM_C206210CA	0.000	2.216	drug induced
Ribose-5-phosphate isomerase (EC 5.3.1.6) (D-ribose-	Q5AJ92	RKI1	CAALFM_C301480CA	0.000	2.216	drug induced
Chitinase 2 (EC 3.2.1.14)	P40953	CHT2	CAALFM_C504130CA	0.000	2.216	drug induced
60S acidic ribosomal protein P1-A (CaRP1A)	Q9HFQ7	RPP1A	CAALFM_C103010WA	0.000	2.216	drug induced
Faa2-3p	A0A1D8PRJ9	FAA2-3	CAALFM_C704180WA	0.000	2.216	drug induced
Ribonuclease P	A0A1D8PDF0	RPM2	CAALFM_C104950CA	0.000	2.216	drug induced
Uncharacterized protein	A0A1D8PQ16	orf19.5578	CAALFM_C602990WA	0.000	2.216	drug induced
Found in mitochondrial proteome protein 38 (Geneti	Q59S77	orf19.3463	CAALFM_C602230WA	0.000	2.216	drug induced
Uncharacterized protein	Q5ANL3	orf19.5890	CAALFM_C304410CA	0.000	3.324	drug induced
Uncharacterized protein	A0A1D8PTF4	orf19.730	CAALFM_CR07190WA	0.000	2.216	drug induced
Uncharacterized protein (Fragment)	A0A1D8PN86	orf19.3202	CAALFM_C501730WA	0.000	2.216	drug induced
Replication factor C subunit 3	A0A1D8PNV1	RFC3	CAALFM_C504000WA	0.000	2.216	drug induced
Putative DNA/RNA helicase	Q5ANG6	SEN1	CAALFM_C304760CA	0.000	2.216	drug induced
Pex11p	A0A1D8PQD7	PEX11	CAALFM_C604310WA	0.000	2.216	drug induced
Lmo1p	A0A1D8PR98	LMO1	CAALFM_C703080WA	0.000	2.216	drug induced

Uncharacterized protein	A0A1D8PLD8	orf19.4626	CAALFM_C401670CA	0.000	2.216	drug induced
Urb2 domain-containing protein	Q5ANL8	orf19.5884	CAALFM_C304370CA	0.000	3.324	drug induced
**Proteins upregulated by DiMIQ**						
Isocitrate lyase	Q59RB8	ICL1	CAALFM_C104500WA	0.911	18.835	-1.305
Fatty acid synthase subunit alpha (EC 2.3.1.86) [Includ	A0A1D8PK65	FAS2	CAALFM_C304830CA	2.734	24.374	-0.976
Sfc1p	A0A1D8PNY6	SFC1	CAALFM_C504440CA	0.911	7.755	-0.958
Dihydroorotate dehydrogenase (quinone), mitochond	Q874I4	URA9	CAALFM_C109720WA	0.911	6.648	-0.898
Acetyl-CoA hydrolase (EC 3.1.2.1) (Acetyl-CoA deacyla	P83773	ACH1	CAALFM_C502000CA	4.556	31.022	-0.871
Phenylalanyl-tRNA synthetase beta subunit (EC 6.1.1.	A0A1D8PS16	FRS1	CAALFM_CR01760CA	0.911	5.540	-0.826
Fatty acid synthase subunit beta (EC 2.3.1.86) [Includ	Q5A4W7	FAS1	CAALFM_C500190CA	4.556	24.374	-0.776
Coproporphyrinogen oxidase (EC 1.3.3.3)	Q59MR4	HEM13	CAALFM_C304060CA	0.911	4.432	-0.739
Gtt11p	A0A1D8PJR2	GTT11	CAALFM_C303720WA	0.911	4.432	-0.739
Translation elongation factor 1 subunit beta	A0A1D8PM35	EFB1	CAALFM_C404480CA	0.911	4.432	-0.739
Sgt2p	Q5A0I8	SGT2	CAALFM_C202830CA	0.911	4.432	-0.739
NAD-specific glutamate dehydrogenase (EC 1.4.1.2)	A0A1D8PI00	GDH2	CAALFM_C207900WA	4.556	18.835	-0.675
Arc1p	A0A1D8PSC8	ARC1	CAALFM_CR03060WA	0.911	3.324	-0.626
Cytochrome c oxidase subunit	A0A1D8PQD5	orf19.1082.1	CAALFM_C604250WA	0.911	3.324	-0.626
Proteasome endopeptidase complex (EC 3.4.25.1)	A0A1D8PRH6	PRE5	CAALFM_C704020CA	0.911	3.324	-0.626
Ribonucleotide-diphosphate reductase subunit	Q5A0L0	RNR21	CAALFM_C203010CA	0.911	3.324	-0.626
Eukaryotic translation initiation factor 3 subunit C (eI	Q5AML1	NIP1	CaO19.12105 CaO19.4635	0.911	3.324	-0.626
Palmitoyltransferase	Q5AI79	YKT6	CAALFM_C102860CA	0.911	3.324	-0.626
Coatomer subunit epsilon	A0A1D8PMT6	orf19.5689	CAALFM_C500080CA	0.911	3.324	-0.626
Signal peptidase complex catalytic subunit SEC11 (EC	Q5A869	SEC11	CAALFM_C401490WA	0.911	3.324	-0.626
Eukaryotic translation initiation factor 4E (eIF-4E) (eIF	Q9P975	TIF45	CAALFM_CR10490WA	0.911	3.324	-0.626
Gcf1p	Q59QB8	GCF1	CAALFM_C108550CA	0.911	3.324	-0.626
FACT complex subunit SPT16 (CaCDC68) (Cell division	Q5A1D5	CDC68	CAALFM_C406500WA	0.911	3.324	-0.626
Phosphoacetylglucosamine mutase (PAGM) (EC 5.4.2	Q5AKW4	AGM1	CAALFM_C113760WA	0.911	3.324	-0.626
Uracil phosphoribosyltransferase	Q59QT3	FUR1	CAALFM_C503390CA	0.911	3.324	-0.626
Phosphoenolpyruvate carboxykinase (ATP) (EC 4.1.1.4	A0A1D8PRM7	PCK1	CAALFM_CR00200WA	5.468	19.942	-0.626
Metalloaminopeptidase	A0A1D8PJE0	LAP41	CAALFM_C302170CA	2.734	8.863	-0.580
Bfr1p	Q5AMT7	BFR1	CAALFM_C402270CA	1.823	5.540	-0.555
Mannose-6-phosphate isomerase (EC 5.3.1.8) (Phosp	P34948	PMI1	CAALFM_C209640WA	1.823	5.540	-0.555
Nucleoside diphosphate kinase (EC 2.7.4.6)	Q5AG68	YNK1	CAALFM_C502890WA	4.556	12.187	-0.505
Copper transport protein CTR1	Q59NP1	CTR1	CAALFM_C600790CA	4.556	12.187	-0.505
Fructose-bisphosphatase (EC 3.1.3.11)	A0A1D8PKW2	FBP1	CAALFM_C307830WA	2.734	6.648	-0.468
Glutamyl-tRNA synthetase (EC 6.1.1.17)	A0A1D8PQL8	GUS1	CAALFM_C700620WA	2.734	6.648	-0.468
Pyrroline-5-carboxylate reductase (EC 1.5.1.2)	A0A1D8PKZ7	PRO3	CAALFM_C400240CA	1.823	4.432	-0.468
ATP synthase subunit delta, mitochondrial (F-ATPase	A0A1D8PUD2	ATP16	CAALFM_CR10850CA	1.823	4.432	-0.468
CCT-alpha (T-complex protein 1 subunit alpha)	Q59QB7	TCP1	CAALFM_C108560WA	1.823	4.432	-0.468
Isoleucine biosynthesis protein	A0A1D8PDX5	MMD1	CAALFM_C106900CA	1.823	4.432	-0.468
Phosphatidylinositol-binding protein	A0A1D8PQC2	orf19.1212	CAALFM_C604100WA	1.823	4.432	-0.468
Mitochondrial import inner membrane translocase su	Q59W44	TIM50	CAALFM_C111220CA	1.823	4.432	-0.468
DNA topoisomerase 2 (EC 5.6.2.2)	A0A1D8PMM1	TOP2	CAALFM_C406600WA	1.823	4.432	-0.468
Alcohol dehydrogenase 2 (EC 1.1.1.1)	O94038	ADH2	CAALFM_C108330CA	5.468	13.295	-0.468
Nuo2p	Q5AEI1	NUO2	CAALFM_C302940CA	0.911	2.216	-0.468
Fox3p	Q5AJ90	FOX3	CAALFM_C301460CA	0.911	2.216	-0.468
Proteasome regulatory particle base subunit	A0A1D8PIV7	RPT2	CAALFM_C300290WA	0.911	2.216	-0.468
Dolichyl-diphosphooligosaccharide--protein glycosylt	Q5AHZ2	orf19.3060	CAALFM_C103600WA	0.911	2.216	-0.468
Alanine transaminase	A0A1D8PJP4	ALT1	CAALFM_C303480CA	0.911	2.216	-0.468
Translation initiation factor eIF5	A0A1D8PNF5	TIF5	CAALFM_C502490CA	0.911	2.216	-0.468
Cytochrome c oxidase subunit 1 (EC 7.1.1.9) (Cytochr	P0C8K9	COX1	CaalfMp08	0.911	2.216	-0.468
Mitochondrial 2-oxodicarboxylate carrier	A0A1D8PT04	orf19.3518	CAALFM_CR05480WA	0.911	2.216	-0.468
Proteasome regulatory particle lid subunit	A0A1D8PNA8	RPN8	CAALFM_C502030WA	0.911	2.216	-0.468
3,4-dihydroxy-2-butanone 4-phosphate synthase (DH	Q5A3V6	RIB3	CAALFM_C112360CA	0.911	2.216	-0.468
RNA polymerase II degradation factor 1	Q5AMM4	DEF1	CAALFM_C401720CA	0.911	2.216	-0.468

Uncharacterized protein	A0A1D8PJE5	orf19.1618.1	CAALFM_C302270WA	0.911	2.216	-0.468
Pdx1p	Q5AKV6	PDX1	CAALFM_C113830CA	0.911	2.216	-0.468
Proteasome regulatory particle base subunit	Q5A2A0	RPT1	CAALFM_C105240CA	0.911	2.216	-0.468
Smi1p	A0A1D8PE53	SMI1	CAALFM_C107870CA	0.911	2.216	-0.468
Cu/Pi carrier	Q59YD1	orf19.1395	CAALFM_C209590CA	0.911	2.216	-0.468
Vesicular-fusion protein SEC18 (EC 3.6.4.6)	A0A1D8PFN6	SEC18	CAALFM_C113580WA	0.911	2.216	-0.468
Hsp90 cochaperone	A0A1D8PQ94	SBA1	CAALFM_C603750CA	0.911	2.216	-0.468
T-complex protein 1 subunit gamma	Q5AK16	CCT3	CAALFM_C505120WA	0.911	2.216	-0.468
CCR4-NOT core subunit	A0A1D8PQD2	CDC39	CAALFM_C604270WA	0.911	2.216	-0.468
Rim1p	Q5AA01	RIM1	CAALFM_C105680CA	0.911	2.216	-0.468
Uncharacterized protein	A0A1D8PNK5	orf19.4332	CAALFM_C503040WA	0.911	2.216	-0.468
Putative hydrolase	Q59WF0	NIT3	CAALFM_C110700CA	0.911	2.216	-0.468
Pheromone-regulated membrane protein 10	Q5AH11	PRM10	CAALFM_C701940CA	0.911	2.216	-0.468
Midasin	A0A1D8PL61	MDN1	CAALFM_C400970CA	0.911	2.216	-0.468
Malate dehydrogenase, cytoplasmic (EC 1.1.1.37)	P83778	MDH1	CAALFM_CR00540CA	8.201	19.942	-0.468
ATP synthase subunit 4, mitochondrial	Q59ZE0	ATP4	CAALFM_C205500WA	5.468	12.187	-0.434
Prolyl-tRNA synthetase (EC 6.1.1.15)	Q59R20	orf19.6701	CAALFM_C703660CA	4.556	9.971	-0.427
Carnitine O-acetyltransferase	Q5AMQ5	CAT2	CAALFM_C402020WA	4.556	9.971	-0.427
Valyl-tRNA synthetase (EC 6.1.1.9)	A0A1D8PHR0	VAS1	CAALFM_C206640CA	3.645	7.755	-0.416
Malate synthase (EC 2.3.3.9)	Q5APD2	MLS1	CAALFM_C109690WA	10.935	23.266	-0.416
HABP4_PAI-RBP1 domain-containing protein	A0A1D8PK71	orf19.5943.1	CAALFM_C304810CA	2.734	5.540	-0.397
Tom40p	Q5AH14	TOM40	CAALFM_C701970CA	2.734	5.540	-0.397
Rab GDP dissociation inhibitor	A0A1D8PFX8	GDI1	CAALFM_C114440CA	2.734	5.540	-0.397
Coronin	A0A1D8PQZ0	CRN1	CAALFM_C701850CA	2.734	5.540	-0.397
Malate dehydrogenase (EC 1.1.1.37)	Q5AMP4	MDH1-1	CAALFM_C401900CA	13.669	27.698	-0.397
Carnitine O-acetyltransferase	Q59MQ8	CTN3	CAALFM_C304000CA	4.556	8.863	-0.381
Isoleucyl-tRNA synthetase (EC 6.1.1.5)	Q59RI1	ILS1	CAALFM_C604520WA	4.556	8.863	-0.381
Bifunctional 2-aminoadipate transaminase/aromatic-A0A1D8PG20		ARO8	CAALFM_C200340CA	4.556	8.863	-0.381
Aspartate transaminase (EC 2.6.1.1)	A0A1D8PHC9	AAT1	CAALFM_C205250CA	10.935	21.050	-0.376
Elongation factor Tu	Q5ABC3	TUF1	CAALFM_C100590WA	6.379	12.187	-0.373
Adenylate kinase (EC 2.7.4.3) (ATP-AMP transphosph	Q5A4Q1	ADK1	CAALFM_C601910WA	8.201	15.511	-0.370
Phospho-2-dehydro-3-deoxyheptonate aldolase, tyroP79023		ARO4	CAALFM_C105110CA	3.645	6.648	-0.355
Cell surface Cu-only superoxide dismutase 5 (EC 1.15	Q5AD07	SOD5	CAALFM_C200680CA	3.645	6.648	-0.355
S-(hydroxymethyl)glutathione dehydrogenase (EC 1.1A0A1D8PU61		FDH3	CAALFM_CR10250CA	3.645	6.648	-0.355
High-affinity glucose transporter 1	A0A1D8PCL1	HGT1	CAALFM_C101980WA	5.468	9.971	-0.355
Oligo-1,6-glucosidase IMA1	A0A1D8PUB9	MAL2	CAALFM_CR10790WA	5.468	9.971	-0.355
Spermidine synthase	Q59Z50	SPE3	CAALFM_C206960WA	1.823	3.324	-0.355
Proteasome endopeptidase complex (EC 3.4.25.1)	A0A1D8PRB6	PUP3	CAALFM_C703390CA	1.823	3.324	-0.355
Oxidoreductase	Q5AML3	orf19.4633	CAALFM_C401510WA	1.823	3.324	-0.355
Ali1p	A0A1D8PJ73	ALI1	CAALFM_C301410CA	1.823	3.324	-0.355
Rho family GTPase	A0A1D8PH96	RHO3	CAALFM_C205030CA	1.823	3.324	-0.355
Type I HSP40 co-chaperone	A0A1D8PSQ3	YDJ1	CAALFM_CR04200WA	1.823	3.324	-0.355
Uncharacterized protein	A0A1D8PFW3	orf19.7234	CAALFM_C114240WA	1.823	3.324	-0.355
RuvB-like helicase 2 (EC 3.6.4.12)	Q5AGZ9	RVB2	CAALFM_C701810WA	1.823	3.324	-0.355
Coatomer subunit delta	A0A1D8PND9	RET2	CAALFM_C502300CA	1.823	3.324	-0.355
CCT-beta	Q59YC4	CCT2	CAALFM_C209520CA	1.823	3.324	-0.355
DUF4149 domain-containing protein	A0A1D8PFM0	orf19.4947	CAALFM_C113190WA	1.823	3.324	-0.355
Uncharacterized protein	A0A1D8PHU4	orf19.2257	CAALFM_C207010WA	1.823	3.324	-0.355
Importin subunit alpha	A0A1D8PMU8	orf19.5682	CAALFM_C500150CA	1.823	3.324	-0.355
Proteasome endopeptidase complex (EC 3.4.25.1)	A0A1D8PJ20	SCL1	CAALFM_C300770CA	1.823	3.324	-0.355
CCT-theta	Q5AB74	CCT8	CAALFM_C100110WA	1.823	3.324	-0.355
Uncharacterized protein	A0A1D8PSE0	orf19.2414	CAALFM_CR03120WA	1.823	3.324	-0.355
Polyamine acetyltransferase	A0A1D8PFY0	orf19.7269	CAALFM_C114500CA	1.823	3.324	-0.355
Glycolipid 2-alpha-mannosyltransferase 1 (EC 2.4.1.-)	Q00310	MNT1	CAALFM_C301810CA	1.823	3.324	-0.355
Acetylglutamate kinase (EC 1.2.1.38) (EC 2.7.2.8) (N-aA0A1D8PEI6		ARG5,6	CAALFM_C109290CA	1.823	3.324	-0.355

Uncharacterized protein	Q5A4Z0	orf19.956	CAALFM_C500330CA	1.823	3.324	-0.355
Multifunctional fusion protein [Includes: L-glutamate	Q5AK46	PUT2	CAALFM_C504880CA	10.024	17.727	-0.343
Biotin carboxylase (EC 6.3.4.14) (EC 6.4.1.2)	A0A1D8PRR7	ACC1	CAALFM_CR00640WA	10.024	17.727	-0.343
Serine--tRNA ligase, cytoplasmic (EC 6.1.1.11) (Seryl-t	Q9HGT6	SES1	CAALFM_C302780WA	8.201	14.403	-0.341
Ato1p	A0A1D8PJ22	ATO1	CAALFM_C300920WA	6.379	11.079	-0.336
Hsp12p (Lipid-binding protein)	A0A1D8PNC7	HSP12	CAALFM_C502080CA	6.379	11.079	-0.336
Agglutinin-like protein 3 (3D9 antigen) (Adhesin 3)	Q59L12	ALS3	CAALFM_CR07070CA	6.379	11.079	-0.336
Polyadenylate-binding protein, cytoplasmic and nucle	Q5AI15	PAB1	CAALFM_C103370WA	6.379	11.079	-0.336
Pyruvate dehydrogenase E1 component subunit beta	Q5A5V6	PDB1	CAALFM_C404150CA	6.379	11.079	-0.336
Hsp90 cochaperone	A0A1D8PN90	STI1	CAALFM_C501820WA	10.935	18.835	-0.333
Protein channel	Q59LZ5	TOM70	CAALFM_C702640WA	9.112	15.511	-0.328
Sbp1p	Q5ANP6	SBP1	CAALFM_C304090WA	4.556	7.755	-0.328
Cytochrome c oxidase subunit Va	Q5APK5	COX5	CAALFM_C109030CA	4.556	7.755	-0.328
Cytochrome c oxidase subunit 2 (EC 7.1.1.9) (Cytochr	Q9B8D8	COX2	CaalfMp01	4.556	7.755	-0.328
NADH-cytochrome b5 reductase 1 (EC 1.6.2.2) (Micro	Q59P03	CBR1	CAALFM_C405450CA	4.556	7.755	-0.328
Peptidyl-prolyl cis-trans isomerase (PPIase) (EC 5.2.1.	Q5ALM6	CPR3	CAALFM_C202320CA	4.556	7.755	-0.328
Citrate synthase	A0A1D8PSH3	CIT1	CAALFM_CR03500WA	32.805	55.396	-0.325
Long-chain fatty acid-CoA ligase	A0A1D8PU56	FAA4	CAALFM_CR10160WA	7.290	12.187	-0.321
5-methyltetrahydropteroyltriglutamate--homocystei	P82610	MET6	CAALFM_CR01620CA	18.225	29.914	-0.314
Branched-chain-amino-acid aminotransferase (EC 2.6	A0A1D8PKB9	BAT22	CAALFM_C305590CA	2.734	4.432	-0.309
ATP synthase subunit e, mitochondrial	A0A1D8PL02	orf19.5660.1	CAALFM_C400330CA	2.734	4.432	-0.309
GTPase-activating protein	A0A1D8PJD7	RNA1	CAALFM_C301990WA	2.734	4.432	-0.309
Uncharacterized protein	Q59WJ7	orf19.2304	CAALFM_C111120CA	2.734	4.432	-0.309
40S ribosomal protein S12	Q5ADQ6	RPS12	CAALFM_C307150CA	2.734	4.432	-0.309
Cytochrome c oxidase subunit (Cytochrome c oxidase	Q5ALV9	COX13	CAALFM_C201590WA	2.734	4.432	-0.309
Glutamate-5-semialdehyde dehydrogenase (EC 1.2.1.	Q5ADR2	PRO2	CAALFM_C307220CA	2.734	4.432	-0.309
Ribosomal protein P2A	A0A1D8PTS0	RPP2A	CAALFM_CR08360CA	2.734	4.432	-0.309
Uncharacterized protein	A0A1D8PCP4	orf19.3684	CAALFM_C102270CA	2.734	4.432	-0.309
Flavodoxin-like fold family protein	A0A1D8PT03	YCP4	CAALFM_CR05380CA	2.734	4.432	-0.309
Carnitine:acyl carnitine antiporter	Q5A967	CRC1	CAALFM_CR01980CA	2.734	4.432	-0.309
Uncharacterized protein	A0A1D8PD97	orf19.5201.1	CAALFM_C104360CA	2.734	4.432	-0.309
Superoxide dismutase (EC 1.15.1.1)	Q5A8Z4	SOD2	CAALFM_C101520CA	2.734	4.432	-0.309
Alpha-ketoglutarate dehydrogenase	A0A1D8PJ26	KGD1	CAALFM_C300880WA	17.314	27.698	-0.304
Isocitrate dehydrogenase [NAD] subunit, mitochondr	A0A1D8PGS5	IDH2	CAALFM_C203080WA	11.846	18.835	-0.302
60S acidic ribosomal protein P0	A0A1D8PQS0	RPP0	CAALFM_C700990WA	11.846	18.835	-0.302
Ornithine aminotransferase (EC 2.6.1.13)	A0A1D8PL14	CAR2	CAALFM_C400160CA	20.959	33.237	-0.301
Formate dehydrogenase (FDH) (EC 1.17.1.9) (NAD-de	Q59QN6	FDH1	CAALFM_CR05170CA	19.136	29.914	-0.295
Isocitrate dehydrogenase [NADP] (EC 1.1.1.42)	A0A1D8PHH7	IDP1	CAALFM_C205890CA	6.379	9.971	-0.295
Heat shock protein 60, mitochondrial (60 kDa chaper	O74261	HSP60	CAALFM_CR06490CA	20.959	32.130	-0.287
Hexokinase-2 (EC 2.7.1.1) (Cytoplasmic antigenic prot	P83776	HXK2	CAALFM_CR04510WA	20.959	32.130	-0.287
Zuotin	Q5AF98	ZUO1	CAALFM_C402870CA	3.645	5.540	-0.284
Thioredoxin reductase (EC 1.8.1.9)	Q5AG89	TRR1	CAALFM_C502710WA	3.645	5.540	-0.284
Tyrosine--tRNA ligase (EC 6.1.1.1) (Tyrosyl-tRNA synth	Q5AFB3	TYS1	CAALFM_C402980WA	3.645	5.540	-0.284
Serine/threonine-protein phosphatase (EC 3.1.3.16)	Q59N42	GLC7	CAALFM_CR07650WA	3.645	5.540	-0.284
Omega-class glutathione transferase	A0A1D8PS56	ECM4	CAALFM_CR02130WA	7.290	11.079	-0.284
Mannose-1-phosphate guanyltransferase (EC 2.7.7.13	O93827	MPG1	CAALFM_C307950CA	8.201	12.187	-0.275
Pyruvate kinase (PK) (EC 2.7.1.40)	P46614	CDC19	CAALFM_C205460WA	21.870	32.130	-0.271
ATP synthase subunit 5, mitochondrial	Q5A7P7	ATP5	CAALFM_C300460WA	9.112	13.295	-0.268
Diphosphomevalonate decarboxylase (EC 4.1.1.33)	A0A1D8PC43	MVD	CAALFM_C100070WA	4.556	6.648	-0.268
Putative phosphotransferase	Q5AF71	orf19.2737	CAALFM_C402620CA	4.556	6.648	-0.268
Cytochrome c oxidase subunit IV	Q5ALV5	COX4	CAALFM_C201620WA	4.556	6.648	-0.268
Arf family GTPase	Q5AND9	ARF2	CAALFM_C304950WA	4.556	6.648	-0.268
Nascent polypeptide-associated complex subunit alp	Q5ANP2	EGD2	CAALFM_C304140CA	4.556	6.648	-0.268
cAMP-dependent protein kinase regulatory subunit (	Q9HEW1	BCY1	CAALFM_C201110CA	4.556	6.648	-0.268
AIR synthase (EC 6.3.3.1) (EC 6.3.4.13) (Glycinamide r	A0A1D8PE67	ADE5,7	CAALFM_C107890CA	4.556	6.648	-0.268

Coatomer subunit alpha	A0A1D8PJB0	orf19.1672	CAALFM_C301720CA	4.556	6.648	-0.268
Dihydrolipoyl dehydrogenase (EC 1.8.1.4)	Q59RQ6	LPD1	CAALFM_CR07400CA	13.669	19.942	-0.268
Acetyl-coenzyme A synthetase 2 (EC 6.2.1.1) (Acetate	Q8NJN3	ACS2	CAALFM_C104290CA	14.580	21.050	-0.264
Succinate--CoA ligase [ADP-forming] subunit beta, mi	A0A1D8PTB5	LSC2	CAALFM_CR06760CA	14.580	21.050	-0.264
Fimbrin	A0A1D8PPY8	SAC6	CAALFM_C602730WA	5.468	7.755	-0.257
ATP synthase subunit d, mitochondrial	Q59PV8	ATP7	CAALFM_C107600WA	5.468	7.755	-0.257
Succinate--CoA ligase [ADP-forming] subunit alpha, m	Q5A8X6	LSC1	CAALFM_C101690CA	5.468	7.755	-0.257
Cytochrome c	P53698	CYC1	CAALFM_C210110WA	5.468	7.755	-0.257
Cam1-1p	A0A1D8PNN8	CAM1-1	CAALFM_C503280WA	6.379	8.863	-0.249
Cys-Gly metallodipeptidase DUG1 (EC 3.4.13.-) (Defic	Q5AKA5	DUG1	CAALFM_C504300CA	6.379	8.863	-0.249
Mitochondrial outer membrane protein porin (Cytop	P83781	POR1	CAALFM_C104100CA	25.515	35.453	-0.249
ATP synthase subunit beta (EC 7.1.2.2)	A0A1D8PKZ9	ATP2	CAALFM_C400270WA	46.473	64.259	-0.247
ATP synthase subunit gamma	A0A1D8PRY3	ATP3	CAALFM_CR01310WA	7.290	9.971	-0.243
Elongation factor 2 (EF-2)	Q5A0M4	EFT2	CAALFM_C203100WA	38.272	52.072	-0.241
Transaldolase (EC 2.2.1.2)	Q5A017	TAL1	CAALFM_CR03720WA	23.692	32.130	-0.239
Succinate dehydrogenase [ubiquinone] flavoprotein s	Q5A1E8	SDH12	CAALFM_C406610CA	8.201	11.079	-0.238
Phosphoglycerate mutase (PGAM) (EC 5.4.2.11) (BPG	P82612	GPM1	CAALFM_C203270WA	10.935	14.403	-0.228
Pyruvate dehydrogenase E1 component subunit alph	Q5A0Z9	PDA1	CAALFM_C407110CA	10.935	14.403	-0.228
Ketol-acid reductoisomerase, mitochondrial (EC 1.1.1	A0A1D8PPG7	ILV5	CAALFM_C600870CA	12.757	16.619	-0.224
Pyridoxal 5'-phosphate synthase (glutamine hydrolyz	Q5AIA6	SNZ1	CAALFM_C102590CA	12.757	16.619	-0.224
ADP/ATP carrier protein	Q5A516	PET9	CAALFM_C500590WA	27.337	35.453	-0.222
5-aminoimidazole-4-carboxamide ribonucleotide form	Q5A6R2	ADE17	CAALFM_CR04090CA	15.491	19.942	-0.219
Fructose-bisphosphate aldolase (FBP aldolase) (FBPA	Q9URB4	FBA1	CAALFM_C401750CA	30.982	38.777	-0.208
Acetyl-coenzyme A synthetase (EC 6.2.1.1)	Q59XW4	ACS1	CAALFM_C210350CA	37.361	46.532	-0.206
**Proteins not affected by DiMIQ**						
Cytochrome b-c1 complex subunit 2, mitochondrial (C	P83782	QCR2	CAALFM_C503350WA	12.757	15.511	-0.197
Mir1p	Q5AP79	MIR1	CAALFM_C110160WA	12.757	15.511	-0.197
Fumarate hydratase (EC 4.2.1.2)	A0A1D8PKV4	FUM12	CAALFM_C307640CA	12.757	15.511	-0.197
Ubiquinol--cytochrome-c reductase subunit	A0A1D8PP59	orf19.4016	CAALFM_C505230CA	15.491	18.835	-0.197
AAA family ATPase	Q59WG3	CDC48	CAALFM_C110790WA	14.580	17.727	-0.197
Coatomer subunit gamma	A0A1D8PDE3	SEC21	CAALFM_C104830WA	4.556	5.540	-0.197
Cytochrome c oxidase subunit VI	A0A1D8PH08	COX6	CAALFM_C203470CA	4.556	5.540	-0.197
(2E,6E)-farnesyl diphosphate synthase (EC 2.5.1.1) (EC	A0A1D8PH78	ERG20	CAALFM_C204580WA	4.556	5.540	-0.197
Protein-synthesizing GTPase (EC 3.6.5.3)	Q5AGF6	GCD11	CAALFM_C502170CA	4.556	5.540	-0.197
Isocitrate dehydrogenase [NADP] (EC 1.1.1.42)	A0A1D8PS79	IDP2	CAALFM_CR02360WA	25.515	31.022	-0.197
MICOS complex subunit MIC60 (Mitofilin)	Q5A044	MIC60	CAALFM_CR03530WA	7.290	8.863	-0.197
DLH domain-containing protein	A0A1D8PLD7	orf19.4609	CAALFM_C401840CA	3.645	4.432	-0.197
Actin-related protein 2	A0A1D8PTX1	ARP2	CAALFM_CR08950WA	3.645	4.432	-0.197
Psa2p	Q5AL34	PSA2	CAALFM_C113160WA	3.645	4.432	-0.197
Pst2p	Q59Y37	PST2	CAALFM_C208640CA	3.645	4.432	-0.197
Regulator of cytoskeleton and endocytosis RVS161	Q5AFE4	RVS161	CAALFM_C700020CA	3.645	4.432	-0.197
Uncharacterized protein	A0A1D8PGJ9	orf19.1549	CAALFM_C202310WA	3.645	4.432	-0.197
Proteasome regulatory particle lid subunit	A0A1D8PLW8	RPN6	CAALFM_C403790WA	3.645	4.432	-0.197
Ran GTPase-binding protein	A0A1D8PES3	NTF2	CAALFM_C110100CA	3.645	4.432	-0.197
Carnitine O-acetyltransferase	Q59T80	CTN1	CAALFM_C101740WA	3.645	4.432	-0.197
26S proteasome regulatory subunit RPN1	A0A1D8PFL7	RPN1	CAALFM_C113300CA	3.645	4.432	-0.197
Asparagine synthase (glutamine-hydrolyzing) (EC 6.3.	A0A1D8PIB2	ASN1	CAALFM_C209060CA	6.379	7.755	-0.197
Calmodulin	A0A1D8PMF8	CMD1	CAALFM_C406030WA	6.379	7.755	-0.197
Thioredoxin	A0A1D8PU69	TRX1	CAALFM_CR10350CA	6.379	7.755	-0.197
Homoserine dehydrogenase (HDH) (EC 1.1.1.3)	Q5AIA2	HOM6	CAALFM_C102620CA	6.379	7.755	-0.197
Small COPII coat GTPase SAR1 (EC 3.6.5.-)	Q59S78	SAR1	CAALFM_C602220WA	6.379	7.755	-0.197
S-adenosylmethionine synthase (EC 2.5.1.6)	A0A1D8PF68	SAM2	CAALFM_C111450CA	16.402	19.942	-0.197
Chorismate synthase (EC 4.2.3.5)	A0A1D8PTK1	ARO2	CAALFM_CR07710WA	2.734	3.324	-0.197
Proliferating cell nuclear antigen	Q5AMN0	POL30	CAALFM_C401770WA	2.734	3.324	-0.197
Protein YOP1	A0A1D8PI76	orf19.2168.3	CAALFM_C208160CA	2.734	3.324	-0.197

Homoisocitrate dehydrogenase	Q5A9D9	LYS12	CAALFM_CR01400WA	2.734	3.324	-0.197
Etr1p	A0A1D8PIW1	ETR1	CAALFM_C300200CA	2.734	3.324	-0.197
Dihydroxyacetone kinase	A0A1D8PEI2	DAK2	CAALFM_C109190CA	2.734	3.324	-0.197
Metalloendopeptidase	Q5A2A7	PRD1	CAALFM_C105300CA	2.734	3.324	-0.197
3-hydroxy-3-methylglutaryl coenzyme A synthase (HMA0A1D8PTW6		ERG13	CAALFM_CR09160CA	2.734	3.324	-0.197
40S ribosomal protein S26	Q5ALV6	RPS26A	CAALFM_C201610CA	2.734	3.324	-0.197
Uncharacterized protein	A0A1D8PTA6	orf19.716	CAALFM_CR06500CA	2.734	3.324	-0.197
Ras-related protein SEC4	P0CY31	SEC4	CAALFM_CR01750CA	2.734	3.324	-0.197
Leucyl-tRNA synthetase (EC 6.1.1.4)	A0A1D8PS12	CDC60	CAALFM_CR01690CA	2.734	3.324	-0.197
Nucleosome assembly protein 1	Q5AAI8	NAP1	CAALFM_CR00320CA	2.734	3.324	-0.197
Cytochrome c oxidase subunit 9, mitochondrial (Cyto A0A1D8PHI5		COX9	CAALFM_C205930WA	2.734	3.324	-0.197
F1F0 ATP synthase subunit i	A0A1D8PG50	ATP18	CAALFM_C200610CA	2.734	3.324	-0.197
DNA polymerase (EC 2.7.7.7)	A0A1D8PK28	POL1	CAALFM_C304300CA	2.734	3.324	-0.197
Guanine nucleotide-binding protein subunit alpha	A0A1D8PJG1	GPA2	CAALFM_C302240CA	2.734	3.324	-0.197
Nucleoporin	A0A1D8PD79	orf19.1054	CAALFM_C104200CA	2.734	3.324	-0.197
PHB domain-containing protein	A0A1D8PTU3	SLP3	CAALFM_CR08990CA	8.201	9.971	-0.197
Glutathione peroxidase	Q59WW7	orf19.86	CAALFM_C600850WA	8.201	9.971	-0.197
Acetyltransferase component of pyruvate dehydrogeQ5AGX8		LAT1	CAALFM_C701640WA	8.201	9.971	-0.197
Ferroxidase	A0A1D8PPC9	FET34	CAALFM_C600440CA	5.468	6.648	-0.197
Yim1p	Q5AHE9	YIM1	CAALFM_C203750WA	5.468	6.648	-0.197
Ecm33p	A0A1D8PCY4	ECM33	CAALFM_C103190CA	13.669	16.619	-0.197
Phosphomannomutase (PMM) (EC 5.4.2.8)	P31353	PMM1	CAALFM_C102480WA	9.112	11.079	-0.197
Phenylalanine--tRNA ligase (EC 6.1.1.20)	A0A1D8PCT4	FRS2	CAALFM_C102710WA	1.823	2.216	-0.197
NAD(P)-bd_dom domain-containing protein	A0A1D8PRL3	orf19.7531	CAALFM_CR00090CA	1.823	2.216	-0.197
Protein transport protein SEC13	Q5AEF2	SEC13	CAALFM_C303170WA	1.823	2.216	-0.197
Acyl carrier protein	Q5AHH7	ACP12	CAALFM_C204030CA	1.823	2.216	-0.197
Dynamin-like GTPase	A0A1D8PN45	VPS1	CAALFM_C501210WA	1.823	2.216	-0.197
Uncharacterized protein	A0A1D8PTU4	orf19.7288	CAALFM_CR08920WA	1.823	2.216	-0.197
Peptidyl-prolyl cis-trans isomerase (PPIase) (EC 5.2.1. A0A1D8PKL0		CYP5	CAALFM_C306360CA	1.823	2.216	-0.197
Rab family GTPase	A0A1D8PTI2	YPT31	CAALFM_CR07520CA	1.823	2.216	-0.197
ANK_REP_REGION domain-containing protein	Q5ANE2	orf19.5961	CAALFM_C304920CA	1.823	2.216	-0.197
6-phosphogluconolactonase-like protein	Q59PZ6	SOL3	CAALFM_CR06700CA	1.823	2.216	-0.197
Isopentenyl-diphosphate Delta-isomerase (EC 5.3.3.2A0A1D8PLI2		IDI1	CAALFM_C402280WA	1.823	2.216	-0.197
Aspartate-semialdehyde dehydrogenase (EC 1.2.1.11 Q5ALM0		HOM2	CAALFM_C202370CA	1.823	2.216	-0.197
Uncharacterized protein	A0A1D8PSW7	orf19.649	CAALFM_CR05030WA	1.823	2.216	-0.197
Proteasome regulatory particle base subunit	A0A1D8PI93	RPT6	CAALFM_C208780WA	1.823	2.216	-0.197
Increased recombination centers protein 22-1	Q59YF4	IRC22-1	CAALFM_C209780CA	1.823	2.216	-0.197
Translationally-controlled tumor protein homolog (TCQ5A860		TMA19	CAALFM_CR00860CA	1.823	2.216	-0.197
Glg2p	A0A1D8PKH2	GLG2	CAALFM_C306450WA	1.823	2.216	-0.197
Non-histone chromosomal protein 6	Q9UVL1	NHP6	CAALFM_C401700CA	1.823	2.216	-0.197
Aha1p	A0A1D8PU72	AHA1	CAALFM_CR10270CA	1.823	2.216	-0.197
Glycine cleavage system H protein	Q5AKX1	GCV3	CAALFM_C113680CA	1.823	2.216	-0.197
Mam33p	A0A1D8PRG4	MAM33	CAALFM_C703930CA	1.823	2.216	-0.197
Yhm1p	A0A1D8PPR4	YHM1	CAALFM_C601930WA	1.823	2.216	-0.197
ATP-dependent RNA helicase DHH1 (EC 3.6.4.13)	Q5AAW3	DHH1	CAALFM_C107070CA	1.823	2.216	-0.197
3-hydroxy-3-methylglutaryl coenzyme A reductase (HA0A1D8PD39		HMG1	CAALFM_C103780CA	1.823	2.216	-0.197
Histone acetyltransferase	A0A1D8PPL1	TRA1	CAALFM_C601320WA	1.823	2.216	-0.197
Proteasome regulatory particle lid subunit	A0A1D8PTV0	RPN7	CAALFM_CR08910CA orf1	1.823	2.216	-0.197
tRNA modification protein	Q5A7B1	MTO1	CAALFM_C107800WA	1.823	2.216	-0.197
Proteasome regulatory particle lid subunit	Q59SD0	RPN12	CAALFM_C208930WA	1.823	2.216	-0.197
Ifg3p	A0A1D8PMX7	IFG3	CAALFM_C500450CA	1.823	2.216	-0.197
Isocitrate dehydrogenase [NAD] subunit, mitochondrA0A1D8PEM5		IDH1	CAALFM_C109630WA	10.024	12.187	-0.197
6-phosphogluconate dehydrogenase, decarboxylatingA0A1D8PFS4		GND1	CAALFM_C113860CA	39.183	45.424	-0.178
Adh1p	A0A1D8PP43	ADH1	CAALFM_C505050WA	18.225	21.050	-0.177
Heat shock protein SSC1, mitochondrial (Cytoplasmic P83784		SSC1	CAALFM_C207380WA	34.627	39.885	-0.176

ATP-dependent RNA helicase eIF4A (EC 3.6.4.13) (Euk	P87206	TIF1	CAALFM_C101350CA	16.402	18.835	-0.174
Aminopeptidase 2 (EC 3.4.11.-)	Q59KZ1	APE2	CAALFM_C104400CA	16.402	18.835	-0.174
Aldehyde dehydrogenase (NAD(P)(+))	A0A1D8PGT5	ALD5	CAALFM_C202970CA	30.982	35.453	-0.173
Inorganic pyrophosphatase (EC 3.6.1.1) (Pyrophospha	P83777	IPP1	CAALFM_C208810CA	12.757	14.403	-0.168
Csp37p	Q5A9D4	CSP37	CAALFM_CR01470WA	10.935	12.187	-0.163
Dihydrolipoyllysine-residue succinyltransferase (EC 2	A0A1D8PTH3	KGD2	CAALFM_CR07420WA	10.935	12.187	-0.163
Aconitate hydratase, mitochondrial (Aconitase) (EC 4	P82611	ACO1	CAALFM_CR08210CA	31.893	35.453	-0.162
Enolase 1 (EC 4.2.1.11) (2-phospho-D-glycerate hydro	P30575	ENO1	CAALFM_C108500CA	61.964	68.691	-0.161
Serine hydroxymethyltransferase, cytosolic (SHMT) (E	O13426	SHM2	CAALFM_C603760CA	10.024	11.079	-0.159
pH-responsive protein 1 (pH-regulated protein 1)	P43076	PHR1	CAALFM_C404530CA	19.136	21.050	-0.157
Superoxide dismutase (EC 1.15.1.1)	A0A1D8PQH5	SOD3	CAALFM_C700110WA	9.112	9.971	-0.155
GTP-binding protein RHO1	O42825	RHO1	CAALFM_CR02860WA	9.112	9.971	-0.155
Aspartyl-tRNA synthetase (EC 6.1.1.12)	Q59UF7	DPS1-1	CAALFM_CR03170WA	9.112	9.971	-0.155
Bifunctional hydroxyacyl-CoA dehydrogenase/enoyl-C	A0A1D8PJ13	FOX2	CAALFM_C300810CA	26.426	28.806	-0.154
Heat shock protein 90 homolog	P46598	HSP90	CAALFM_C702030WA	42.828	46.532	-0.153
Aspartate carbamoyltransferase (EC 2.1.3.2) (EC 6.3.5	A0A1D8PTD1	URA2	CAALFM_CR07050CA	16.402	17.727	-0.151
Protein SUR7	Q5A4M8	SUR7	CAALFM_C601720CA	8.201	8.863	-0.151
Adenosylhomocysteinase (AdoHcyase) (EC 3.3.1.1) (S	P83783	SAH1	CAALFM_C504270CA	15.491	16.619	-0.148
Heat shock protein SSA1	P41797	SSA1	CAALFM_C113480WA	101.150	107.470	-0.144
Thioredoxin peroxidase	Q5AF44	AHP1	CAALFM_C402410CA	13.669	14.403	-0.141
RRM domain-containing protein	Q5AK88	orf19.3932	CAALFM_C504470CA	13.669	14.403	-0.141
ATP synthase subunit alpha	A0A1D8PDC4	ATP1	CAALFM_C104610WA	47.385	49.856	-0.140
Eukaryotic translation initiation factor 5A (eIF-5A) (eI	O94083	ANB1	CAALFM_C601610WA	6.379	6.648	-0.136
Diadenosine tetraphosphate synthetase (EC 6.1.1.14)	Q5A2A5	GRS1	CAALFM_C105290WA	6.379	6.648	-0.136
Tubulin beta chain	A0A1D8PC97	TUB2	CAALFM_C100710CA	19.136	19.942	-0.136
Chaperone ATPase	A0A1D8PTP9	HSP104	CAALFM_CR08250CA	35.538	36.561	-0.131
Hsp70 family ATPase	Q5A397	SSB1	CAALFM_CR08090WA	41.006	42.101	-0.131
Putative NADPH-dependent methylglyoxal reductase	P83775	GRP2	CAALFM_C502860CA	17.314	17.727	-0.129
Triosephosphate isomerase (TIM) (EC 5.3.1.1) (Triose	Q9P940	TPI1	CAALFM_C307440WA	16.402	16.619	-0.125
Pho88p	A0A1D8PTX9	PHO88	CAALFM_CR09320CA	5.468	5.540	-0.125
Profilin	Q5A786	PFY1	CAALFM_C108030WA	5.468	5.540	-0.125
Cytochrome b-c1 complex subunit 7	Q5ABS1	QCR7	CAALFM_C603400CA	5.468	5.540	-0.125
Corticosteroid-binding protein	P31225	CBP1	CAALFM_CR09270CA	10.935	11.079	-0.125
Putative ammonium permease	A0A1D8PHP8	FRP3	CAALFM_C206680WA	10.935	11.079	-0.125
Endoplasmic reticulum chaperone BiP (EC 3.6.4.10) (I	A0A1D8PG96	KAR2	CAALFM_C201120WA	37.361	37.669	-0.123
Protein disulfide-isomerase (EC 5.3.4.1)	A0A1D8PR99	PDI1	CAALFM_C703250CA	15.491	15.511	-0.121
Peroxiredoxin TSA1-B (EC 1.11.1.24) (Thiol-specific an	P0CU34	TSA1B	CAALFM_C306330WA	40.095	39.885	-0.118
L-iditol 2-dehydrogenase	A0A1D8PUB4	XYL2	CAALFM_CR10840CA	10.024	9.971	-0.118
Guanine nucleotide-binding protein subunit beta-like	P83774	ASC1	CAALFM_C701250WA	10.024	9.971	-0.118
Protein transport protein SEC24	Q5AQ76	SEC24	CAALFM_C108740CA	10.024	9.971	-0.118
NADPH--cytochrome P450 reductase (CPR) (P450R) (E	A0A1D8PLR7	NCP1	CAALFM_C403180WA	4.556	4.432	-0.109
Saccharopine dehydrogenase [NAD(+), L-lysine-formi	P43065	LYS1	CAALFM_C405320WA	4.556	4.432	-0.109
Glucose-6-phosphate 1-epimerase (EC 5.1.3.15)	Q5A1Q0	orf19.1946	CAALFM_C501230CA	4.556	4.432	-0.109
Ssz1p	Q5A678	SSZ1	CAALFM_C404700WA	4.556	4.432	-0.109
Hexose transporter	Q5AD47	HGT6	CAALFM_C201020WA	4.556	4.432	-0.109
GMP synthase [glutamine-hydrolyzing] (EC 6.3.5.2) (G	Q5APF2	GUA1	CAALFM_C109490CA	4.556	4.432	-0.109
Uncharacterized protein	Q5A8Z9	orf19.3335	CAALFM_C101490WA	4.556	4.432	-0.109
Uncharacterized protein	A0A1D8PFU8	orf19.7215.3	CAALFM_C114090WA	4.556	4.432	-0.109
Uncharacterized protein	A0A1D8PQ38	orf19.5597.1	CAALFM_C603130WA	4.556	4.432	-0.109
Saccharopine dehydrogenase (NADP+, L-glutamate-fo	A0A1D8PKJ4	LYS9	CAALFM_C306590WA	9.112	8.863	-0.109
Actin	A0A1D8PFR4	ACT1	CAALFM_C113700WA	54.675	53.180	-0.109
Transketolase (EC 2.2.1.1)	Q5A750	TKL1	CAALFM_C108320WA	20.959	19.942	-0.101
Pyruvate carboxylase (EC 6.4.1.1)	A0A1D8PLY4	PYC2	CAALFM_C403940CA	20.959	19.942	-0.101
Aspartate aminotransferase (EC 2.6.1.1)	Q59N40	AAT21	CAALFM_CR07620WA	8.201	7.755	-0.098
Protein transport protein SEC31	Q5AAU3	PGA63	CAALFM_C106930WA	8.201	7.755	-0.098

Alpha,alpha-trehalose-phosphate synthase [UDP-form	Q92410	TPS1	CAALFM_CR05720WA	8.201	7.755	-0.098
V-type proton ATPase catalytic subunit A (V-ATPase s	Q5AJB1	TFP1	CAALFM_C301630WA	24.604	23.266	-0.098
Mrf1p	Q59TU5	MRF1	CAALFM_C111700CA	11.846	11.079	-0.094
Pyruvate decarboxylase (EC 4.1.1.1)	P83779	PDC11	CAALFM_C406570CA	51.030	47.640	-0.093
14-3-3 protein homolog	O42766	BMH1	CAALFM_C103220CA	22.781	21.050	-0.089
Septin	A0A1D8PCY5	CDC12	CAALFM_C103210CA	7.290	6.648	-0.084
40S ribosomal protein S8	Q59T44	RPS8A	CAALFM_C205610CA	7.290	6.648	-0.084
Bifunctional cysteine synthase/O-acetylhomoserine a	Q59US5	MET15	CAALFM_C400200CA	7.290	6.648	-0.084
Ribosomal protein L15	Q5A6R1	RPL15A	CAALFM_CR04100CA	7.290	6.648	-0.084
Peptidyl-prolyl cis-trans isomerase D (PPIase D) (EC 5	Q5ACI8	CPR6	CAALFM_CR10670WA	7.290	6.648	-0.084
Tfs1p	Q5A1M1	TFS1	CAALFM_C500930CA	7.290	6.648	-0.084
Obg-like ATPase 1	A0A1D8PDE8	YBN5	CAALFM_C104890WA	7.290	6.648	-0.084
Uncharacterized protein	A0A1D8PCU5	orf19.2954	CAALFM_C102650WA	3.645	3.324	-0.084
Phospho-2-dehydro-3-deoxyheptonate aldolase (EC 2	A0A1D8PGI8	ARO3	CAALFM_C202030WA	3.645	3.324	-0.084
Asparaginyl-tRNA synthetase (EC 6.1.1.22)	Q59R18	DED81	CAALFM_C703670WA	3.645	3.324	-0.084
Ribosomal 60S subunit protein L30	A0A1D8PM75	RPL30	CAALFM_C404900WA	3.645	3.324	-0.084
1,3-beta-glucanosyltransferase PGA4 (EC 2.4.1.-) (GPI	Q5AJY5	PGA4	CAALFM_C505390CA	3.645	3.324	-0.084
Prohibitin	A0A1D8PJT2	orf19.357	CAALFM_C303590WA	3.645	3.324	-0.084
Ribosomal 60S subunit protein L24A	Q5A6A1	RPL24A	CAALFM_C404890CA	3.645	3.324	-0.084
Ribosomal 60S subunit protein L25	A0A1D8PPS1	RPL25	CAALFM_C601970CA	3.645	3.324	-0.084
SBDS domain-containing protein	Q59U89	orf19.1862	CAALFM_C207630CA	3.645	3.324	-0.084
Pst1p	A0A1D8PHR5	PST1	CAALFM_C206870CA	3.645	3.324	-0.084
Rct1p	A0A1D8PKD1	RCT1	CAALFM_C305710WA	3.645	3.324	-0.084
Cytochrome b-c1 complex subunit Rieske, mitochond	A0A1D8PJX3	RIP1	CAALFM_C304430WA	3.645	3.324	-0.084
FACT complex subunit POB3 (Facilitates chromatin tra	Q5ALL8	POB3	CAALFM_C202380WA	3.645	3.324	-0.084
Phosphotransferase (EC 2.7.1.-)	Q59TZ8	GLK1	CAALFM_CR07150WA	21.870	19.942	-0.084
Peptidyl-prolyl cis-trans isomerase (PPIase) (EC 5.2.1.	P22011	CYP1	CAALFM_C702380CA	17.314	15.511	-0.077
Phosphoglucomutase	A0A1D8PSA9	PGM2	CAALFM_CR02820WA	13.669	12.187	-0.075
NADH-cytochrome b5 reductase 2 (EC 1.6.2.2) (Mitoc	Q59M70	MCR1	CAALFM_C602040WA	13.669	12.187	-0.075
Ubiquitin-ribosomal 40S subunit protein S31 fusion p	Q5A109	UBI3	CAALFM_C407180WA	10.024	8.863	-0.072
ATP-dependent 6-phosphofructokinase (ATP-PFK) (Ph	Q5AGZ8	PFK2	CAALFM_C701800CA	10.024	8.863	-0.072
Rdi1p	Q5AND4	RDI1	CAALFM_C305000WA	10.024	8.863	-0.072
Glucose-6-phosphate 1-dehydrogenase (EC 1.1.1.49)	A0A1D8PEG2	ZWF1	CAALFM_C108980CA	16.402	14.403	-0.069
Tubulin alpha chain	A0A1D8PTV4	TUB1	CAALFM_CR09120CA	16.402	14.403	-0.069
Proteinase A	Q59U59	APR1	CAALFM_C207400CA	6.379	5.540	-0.065
Protein transport protein SEC23	Q5A455	SEC23	CAALFM_C405690WA	6.379	5.540	-0.065
Uncharacterized protein	A0A1D8PQ54	orf19.5620	CAALFM_C603320WA	6.379	5.540	-0.065
Glucose-6-phosphate isomerase (GPI) (EC 5.3.1.9) (Ph	P83780	PGI1	CAALFM_CR06340CA	34.627	29.914	-0.063
Formyltetrahydrofolate synthetase (EC 1.5.1.5) (EC 3.	Q59SM8	MIS11	CAALFM_CR07010WA	21.870	18.835	-0.062
Translation elongation factor EF1B gamma	A0A1D8PKC3	CAM1	CAALFM_C306010WA	15.491	13.295	-0.060
Csh1p	Q59QH2	CSH1	CAALFM_C104020CA	40.095	34.345	-0.060
Lipid-binding protein	Q59KV8	LSP1	CAALFM_C206730WA	33.716	28.806	-0.059
Slk19p	Q5ADT0	SLK19	CAALFM_C307310CA	30.071	25.482	-0.055
Septin	A0A1D8PD83	CDC3	CAALFM_C104210CA	11.846	9.971	-0.053
Alpha-1,4 glucan phosphorylase (EC 2.4.1.1)	A0A1D8PQQ3	GPH1	CAALFM_C700930WA	42.828	35.453	-0.046
V-type proton ATPase subunit B (V-ATPase subunit B)	Q59PT0	VMA2	CAALFM_CR05780WA	25.515	21.050	-0.045
Elongation factor 3 (EF-3)	P25997	CEF3	CAALFM_C501580CA	28.249	23.266	-0.044
Phosphoglycerate kinase (EC 2.7.2.3)	P46273	PGK1	CAALFM_C600750CA	71.988	58.719	-0.040
**Proteins downregulated by DiMIQ**						
Glyoxalase 3 (EC 4.2.1.130) (Glutathione-independen	Q5AF03	GLX3	CAALFM_C302610CA	38.272	31.022	-0.038
Actin-depolymerizing factor 1 (Cofilin)	A0A1D8PMW6	COF1	CAALFM_C500370WA	5.468	4.432	-0.038
Het1p	A0A1D8PPA5	HET1	CAALFM_C600100CA	5.468	4.432	-0.038
Adenylosuccinate lyase (ASL) (EC 4.3.2.2) (Adenylosuc	A0A1D8PT56	ADE13	CAALFM_CR06150CA	5.468	4.432	-0.038
Uncharacterized protein	A0A1D8PU51	orf19.7590	CAALFM_CR10140WA	5.468	4.432	-0.038
Cystathionine beta-synthase (EC 4.2.1.22)	Q59T95	CYS4	CAALFM_C101870CA	5.468	4.432	-0.038

Glucosamine_iso domain-containing protein	Q59YH1	orf19.1355	CAALFM_C209970CA	5.468	4.432	-0.038
Rab family GTPase	Q5AI00	YPT1	CAALFM_C103500WA	5.468	4.432	-0.038
Hyphally regulated cell wall protein 1 (Adhesin-like p	Q5AL03	HYR1	CAALFM_C113450WA	5.468	4.432	-0.038
Ifr2p	A0A1D8PSE7	IFR2	CAALFM_CR03280WA	5.468	4.432	-0.038
Formylglycinamide ribonucleotide amidotransferase	Q59MZ5	ADE6	CAALFM_CR04740CA	5.468	4.432	-0.038
Karyopherin beta	Q59VX7	orf19.3681	CAALFM_C102240WA	2.734	2.216	-0.038
S-formylglutathione hydrolase (EC 3.1.2.12)	A0A1D8PU04	orf19.6596	CAALFM_CR09670CA	2.734	2.216	-0.038
Hsp70 family chaperone	A0A1D8PGU0	LHS1	CAALFM_C202760WA	2.734	2.216	-0.038
Ran GTPase-binding protein	A0A1D8PRQ3	YRB1	CAALFM_CR00580WA	2.734	2.216	-0.038
Translation initiation factor eIF2 subunit alpha	Q5AAU7	SUI2	CAALFM_C106960WA	2.734	2.216	-0.038
Septation protein 7 (Seventh homolog of septin 1)	Q59VX8	SEP1	CAALFM_C102230WA	2.734	2.216	-0.038
Dolichyl-diphosphooligosaccharide--protein glycosylt	A0A1D8PK87	OST1	CAALFM_C305530WA	2.734	2.216	-0.038
Very-long-chain 3-oxoacyl-CoA reductase (EC 1.1.1.33	Q59V93	orf19.3859	CAALFM_CR06070WA	2.734	2.216	-0.038
Snl1p	Q59NB3	SNL1	CAALFM_C110530WA	2.734	2.216	-0.038
Inosine-5'-monophosphate dehydrogenase (IMP deh	Q59Q46	IMH3	CAALFM_C206390CA	2.734	2.216	-0.038
WD_REPEATS_REGION domain-containing protein	A0A1D8PSI3	orf19.4395	CAALFM_CR03520CA	2.734	2.216	-0.038
Prohibitin	Q5AND0	PHB2	CAALFM_C305030WA	2.734	2.216	-0.038
Wh11p	A0A1D8PHF8	WH11	CAALFM_C205180WA	2.734	2.216	-0.038
Ubiquinol--cytochrome-c reductase subunit 8	A0A1D8PHA2	QCR8	CAALFM_C204590CA	2.734	2.216	-0.038
NAD(P)-bd_dom domain-containing protein	A0A1D8PJA6	orf19.1682	CAALFM_C301610WA	2.734	2.216	-0.038
Dolichyl-phosphate-mannose--protein mannosyltran	O74189	PMT1	CAALFM_C702890CA	2.734	2.216	-0.038
Ribosomal 60S subunit protein L5	Q5AGZ7	RPL5	CAALFM_C701790CA	2.734	2.216	-0.038
Thioredoxin peroxidase	Q5A7P9	DOT5	CAALFM_C300480CA	2.734	2.216	-0.038
Phosphoribosylaminoimidazole-succinocarboxamide	A0A1D8PRQ1	ADE1	CAALFM_CR00510CA	2.734	2.216	-0.038
NADH-ubiquinone reductase (H(+)-translocating)	Q5AEC9	NDE1	CAALFM_C303420CA	2.734	2.216	-0.038
Septin CDC11 (Cell division control protein 11)	G1UB61	CDC11	CAALFM_C500070WA	2.734	2.216	-0.038
Coatomer subunit beta (Beta-coat protein)	Q5A6M6	SEC26	CAALFM_CR04380CA	2.734	2.216	-0.038
Trifunctional aldehyde reductase/xylose reductase/g	A0A1D8PNK3	GRE3	CAALFM_C502930CA	10.935	8.863	-0.038
Glutamate decarboxylase (EC 4.1.1.15)	A0A1D8PF79	GAD1	CAALFM_C111660WA	24.604	19.942	-0.038
Glyceraldehyde-3-phosphate dehydrogenase (EC 1.2.	Q5ADM7	TDH3	CAALFM_C306870WA	165.850	131.840	-0.030
E1 ubiquitin-activating protein	A0A1D8PKJ3	UBA1	CAALFM_C306500WA	12.757	9.971	-0.024
Ifd6p	A0A1D8PD78	IFD6	CAALFM_C104140WA	25.515	19.942	-0.024
Aldehyde dehydrogenase	A0A1D8PC76	orf19.6066	CAALFM_C100410CA	10.024	7.755	-0.020
Tropomyosin	A0A1D8PTR7	TPM2	CAALFM_CR08460WA	10.024	7.755	-0.020
Type II HSP40 co-chaperone	Q59V92	SIS1	CAALFM_CR06080WA	10.024	7.755	-0.020
Lysine--tRNA ligase (EC 6.1.1.6) (Lysyl-tRNA synthetas	Q5ADU2	KRS1	CAALFM_C307410CA	10.024	7.755	-0.020
Elongation factor 1-alpha 1 (EF-1-alpha 1)	P0CY35	TEF1	CAALFM_C208370CA	74.722	57.612	-0.018
Acetyl-CoA C-acetyltransferase	A0A1D8PH52	ERG10	CAALFM_C204310WA	17.314	13.295	-0.017
Ras family GTPase	A0A1D8PS50	RSR1	CAALFM_CR02140WA	7.290	5.540	-0.013
Succinate dehydrogenase [ubiquinone] iron-sulfur su	Q59QN7	SDH2	CAALFM_CR05180CA	7.290	5.540	-0.013
Adenylyl cyclase-associated protein	Q5A6P9	SRV2	CAALFM_CR04190WA	7.290	5.540	-0.013
40S ribosomal protein S24	Q5A7K0	RPS24	CAALFM_C300090WA	7.290	5.540	-0.013
Aldo_ket_red domain-containing protein	A0A1D8PD74	orf19.4476	CAALFM_C104010CA	21.870	16.619	-0.013
Heat shock protein 78, mitochondrial	Q96UX5	HSP78	CAALFM_C203390CA	21.870	16.619	-0.013
Uncharacterized protein	A0A1D8PF42	orf19.2296	CAALFM_C111200WA	26.426	19.942	-0.010
ATP-dependent RNA helicase	A0A1D8PKZ3	SUB2	CAALFM_C400220CA	11.846	8.863	-0.007
Reticulon-like protein	A0A1D8PM53	orf19.3799	CAALFM_C404800WA	11.846	8.863	-0.007
Clathrin heavy chain (Fragment)	A0A1D8PPS9	CHC1	CAALFM_C602120WA	16.402	12.187	-0.004
Plasma membrane ATPase (EC 7.1.2.1)	A0A1D8PJ01	PMA1	CAALFM_C300720WA	65.609	48.748	-0.004
Lipid-binding protein	A0A1D8PDD1	PIL1	CAALFM_C104680WA	32.805	24.374	-0.004
Glycogen [starch] synthase (EC 2.4.1.11)	Q5A850	GSY1	CAALFM_CR00780CA	9.112	6.648	0.003
Ribosomal 40S subunit protein S5	Q5AG43	RPS5	CAALFM_C503070WA	9.112	6.648	0.003
Threonine synthase (EC 4.2.3.1)	A0A1D8PNG9	THR4	CAALFM_C502270WA	4.556	3.324	0.003
Riboflavin synthase	A0A1D8PP67	RIB5	CAALFM_C505300WA	4.556	3.324	0.003
Peptidase_M24 domain-containing protein	A0A1D8PR11	orf19.6507	CAALFM_C702100WA	4.556	3.324	0.003

Carboxypeptidase (EC 3.4.16.-)	A0A1D8PRC2	CPY1	CAALFM_C703360WA	4.556	3.324	0.003
Uncharacterized protein	A0A1D8PT83	orf19.715	CAALFM_CR06510WA	4.556	3.324	0.003
Cell division control protein 10	P39827	CDC10	CAALFM_CR04570CA	4.556	3.324	0.003
Malate dehydrogenase (EC 1.1.1.37)	Q5A5S6	MDH1-3	CAALFM_C210480WA	4.556	3.324	0.003
SCP2 domain-containing protein	Q5AJ84	orf19.1709	CAALFM_C301420CA	4.556	3.324	0.003
Ribosomal 60S subunit protein L22B	A0A1D8PM41	orf19.1409.1	CAALFM_C404390WA	4.556	3.324	0.003
Asr3p	A0A1D8PH00	ASR3	CAALFM_C203790CA	4.556	3.324	0.003
Heat shock protein homolog SSE1 (Chaperone protein	Q96VB9	MSI3	CAALFM_C106100CA	27.337	19.942	0.003
Ras-like protein 1 (Ras homolog type B)	Q59XU5	RAS1	CAALFM_C210210CA	13.669	9.971	0.003
Mdg1p	A0A1D8PFV7	MDG1	CAALFM_C114290CA	24.604	17.727	0.008
Homocitrate synthase (EC 2.3.3.14)	Q59TC4	LYS22	CAALFM_C204460WA	10.935	7.755	0.014
Phm7p	A0A1D8PI19	PHM7	CAALFM_C208140CA	17.314	12.187	0.017
40S ribosomal protein S4	A0A1D8PCI6	RPS42	CAALFM_C101640WA	12.757	8.863	0.022
Ribosomal 40S subunit protein S16A	A0A1D8PCW6	RPS16A	CAALFM_C103030WA	6.379	4.432	0.022
Endoplasmic reticulum transmembrane protein	A0A1D8PGL1	orf19.1564	CAALFM_C202410WA	6.379	4.432	0.022
Ribosomal 60S subunit protein L11B	A0A1D8PHW1	RPL11	CAALFM_C206810CA	6.379	4.432	0.022
Adenosine kinase (EC 2.7.1.20)	A0A1D8PQ26	ADO1	CAALFM_C603080CA	6.379	4.432	0.022
Transcriptional repressor TUP1	P0CY34	TUP1	CAALFM_C100060WA	6.379	4.432	0.022
Alanine--tRNA ligase (EC 6.1.1.7) (Alanyl-tRNA synthe	Q5A8K2	ALA1	CAALFM_C603720WA	6.379	4.432	0.022
Inhibitor I9 domain-containing protein	Q5AF37	orf19.2769	CAALFM_C402340WA	6.379	4.432	0.022
Non-classical export protein 102	Q5ANE3	NCE102	CAALFM_C304910CA	6.379	4.432	0.022
High-affinity iron permease	A0A1D8PFV0	FTR1	CAALFM_C114130WA	6.379	4.432	0.022
Uncharacterized protein	A0A1D8PD11	orf19.3053	CAALFM_C103510CA	8.201	5.540	0.033
Ribosomal 60S subunit protein L2A	A0A1D8PF08	RPL2	CAALFM_C111060CA	8.201	5.540	0.033
40S ribosomal protein S0	O42817	RPS0	CAALFM_C305370CA	8.201	5.540	0.033
Ribosomal 60S subunit protein L9B	Q5AEN2	RPL9B	CAALFM_C302470CA	8.201	5.540	0.033
Stress protein DDR48 (DNA damage-responsive prote	Q59X49	DDR48	CAALFM_C209220WA	8.201	5.540	0.033
Uncharacterized protein	A0A1D8PJ10	orf19.6160	CAALFM_C300850CA	18.225	12.187	0.037
Pleiotropic ABC efflux transporter of multiple drugs C	Q5ANA3	CDR1	CAALFM_C305220WA	18.225	12.187	0.037
pH-regulated antigen PRA1 (58 kDa fibrinogen-bindin	P87020	PRA1	CAALFM_C406980WA	28.249	18.835	0.038
Abp1p	Q5AFA8	ABP1	CAALFM_C402940WA	10.024	6.648	0.041
17-beta-hydroxysteroid dehydrogenase-like protein	A0A1D8PFV8	orf19.7214	CAALFM_C114060WA	11.846	7.755	0.046
Uncharacterized protein	A0A1D8PF90	orf19.5281	CAALFM_C111860WA	7.290	4.432	0.075
Flavodoxin-like fold family protein	A0A1D8PT02	PST3	CAALFM_CR05390WA	7.290	4.432	0.075
V-type proton ATPase subunit a	Q59R99	VPH1	CAALFM_C405240CA	7.290	4.432	0.075
D-3-phosphoglycerate dehydrogenase (EC 1.1.1.399)	Q5A3K7	SER33	CAALFM_C112030WA	7.290	4.432	0.075
Small heat shock protein 21	Q5AHH4	HSP21	CAALFM_C204010CA	7.290	4.432	0.075
Dolichol-phosphate mannosyltransferase subunit 1 (EA	A0A1D8PEA2	DPM1	CAALFM_C108010WA	9.112	5.540	0.075
Uncharacterized protein	A0A1D8PQJ8	orf19.7085	CAALFM_C700350CA	9.112	5.540	0.075
GTP-binding nuclear protein	Q59P43	GSP1	CAALFM_C206310CA	10.935	6.648	0.075
Ribosomal 60S subunit protein L3	Q59LS1	RPL3	CAALFM_C209430WA	14.580	8.863	0.075
D-arabinose 1-dehydrogenase (NAD(P)(+))	A0A1D8PI24	ARA1	CAALFM_C208130WA	5.468	3.324	0.075
1,4-alpha-glucan-branching enzyme (EC 2.4.1.18) (Gly	A0A1D8PQ59	GLC3	CAALFM_C603340CA	5.468	3.324	0.075
Ribosomal 60S subunit protein L12A	Q5AJF7	RPL12	CAALFM_C302110WA	5.468	3.324	0.075
Adenylosuccinate synthetase (AMPSase) (AdSS) (EC 6	P0CH96	ADE12	CAALFM_C109640WA	5.468	3.324	0.075
V-type proton ATPase subunit	A0A1D8PKX3	VMA6	CAALFM_C400020WA	5.468	3.324	0.075
Transcriptional regulator HMO1 (High mobility group	Q59PR9	HMO1	CAALFM_CR05670CA	5.468	3.324	0.075
Leu42p	A0A1D8PIF8	LEU42	CAALFM_C209750WA	3.645	2.216	0.075
Long-chain fatty acid transporter	A0A1D8PQN3	ACB1	CAALFM_C700750WA	3.645	2.216	0.075
Proteinase B	A0A1D8PRH0	orf19.7196	CAALFM_C703860WA	3.645	2.216	0.075
Uncharacterized protein	A0A1D8PMU2	orf19.5686	CAALFM_C500100CA	3.645	2.216	0.075
40S ribosomal protein S25	A0A1D8PNQ6	RPS25B	CAALFM_C503540CA	3.645	2.216	0.075
Erp5p	A0A1D8PF48	ERP5	CAALFM_C110940CA	3.645	2.216	0.075
Ribosomal 60S subunit protein L21A	A0A1D8PGY0	RPL21A	CAALFM_C203810CA	3.645	2.216	0.075
Rho family GTPase	A0A1D8PDV5	RAC1	CAALFM_C106730WA	3.645	2.216	0.075

40S ribosomal protein S27	A0A1D8PTI7	RPS27	CAALFM_CR07630CA	3.645	2.216	0.075
F1F0 ATP synthase subunit h	A0A1D8PHL7	ATP14	CAALFM_C206290CA	3.645	2.216	0.075
Cation-transporting ATPase (EC 7.2.2.-)	Q59Q34	SPF1	CAALFM_C206540CA	3.645	2.216	0.075
Protein transport protein SEC61 subunit alpha	Q9P8E3	SEC61	CAALFM_C307810CA	3.645	2.216	0.075
Arginyl-tRNA synthetase (EC 6.1.1.19)	A0A1D8PCI5	orf19.3341	CAALFM_C101530CA	3.645	2.216	0.075
26S proteasome regulatory subunit RPN2	Q5A3L0	RPN2	CAALFM_C112050WA	1.823	1.108	0.075
Uncharacterized protein	A0A1D8PPT3	orf19.3499	CAALFM_C602100WA	1.823	1.108	0.075
Kre9p	Q5ANN9	KRE9	CAALFM_C304180WA	1.823	1.108	0.075
Ribosomal 60S subunit protein L35A	A0A1D8PK30	RPL35	CAALFM_C304960WA	1.823	1.108	0.075
Endoplasmic reticulum vesicle protein 25	Q5A302	ERV25	CAALFM_C306250WA	1.823	1.108	0.075
Lhp1p	A0A1D8PE36	LHP1	CAALFM_C107500CA	1.823	1.108	0.075
Proteasome regulatory particle lid subunit	A0A1D8PGA6	RPN9	CAALFM_C201320WA	1.823	1.108	0.075
Mitogen-activated protein kinase HOG1 (MAP kinase	Q92207	HOG1	CAALFM_C203330CA	1.823	1.108	0.075
GPI-anchored protein 52	Q59L72	PGA52	CAALFM_C200100CA	1.823	1.108	0.075
Vacuolar calcium ion transporter	Q59QB4	VCX1	CAALFM_C108580CA	1.823	1.108	0.075
Ribosomal 60S subunit protein L34B	A0A1D8PDZ1	orf19.6220.4	CAALFM_C106890CA	1.823	1.108	0.075
Translation initiation factor eIF4G	A0A1D8PI73	TIF4631	CAALFM_C208760CA	1.823	1.108	0.075
Lysophospholipase (EC 3.1.1.5)	A0A1D8PU17	PLB3	CAALFM_CR09690CA	1.823	1.108	0.075
B30.2/SPRY domain-containing protein	A0A1D8PRG7	orf19.7193	CAALFM_C703890CA	1.823	1.108	0.075
Coatomer subunit beta'	A0A1D8PPV5	SEC27	CAALFM_C602260CA	1.823	1.108	0.075
Ornithine transcarbamylase	Q5ABU0	ARG3	CAALFM_C603230WA	1.823	1.108	0.075
Candidapepsin-9 (EC 3.4.23.24) (ACP 9) (Aspartate pro	Q59SU1	SAP9	CAALFM_C303870CA	1.823	1.108	0.075
Protein transport protein Sec61 subunit beta	A0A1D8PRY9	orf19.2533.1	CAALFM_CR01490CA	1.823	1.108	0.075
Actin-regulating kinase PRK1 (EC 2.7.11.1)	Q5A961	PRK1	CAALFM_CR02040WA	1.823	1.108	0.075
Fumarate reductase (EC 1.3.1.6)	Q59T35	OSM1	CAALFM_C205700WA	1.823	1.108	0.075
Medium-chain fatty acid-CoA ligase	A0A1D8PJI7	FAA21	CAALFM_C302810CA	1.823	1.108	0.075
HECT-type E3 ubiquitin transferase (EC 2.3.2.26)	A0A1D8PGT3	TOM1	CAALFM_C203180CA	1.823	1.108	0.075
Mlc1p	A0A1D8PSE1	MLC1	CAALFM_CR03090CA	1.823	1.108	0.075
Transcription factor TFIIIC subunit	A0A1D8PQW6	orf19.6559	CAALFM_C701650WA	1.823	1.108	0.075
Chaperonin-containing T-complex subunit	A0A1D8PMN9	CCT6	CAALFM_C406830CA	1.823	1.108	0.075
Mitochondrial intermembrane space import and asse	O94030	MIA40	CAALFM_C102880CA	1.823	1.108	0.075
Histone-glutamine methyltransferase (rRNA 2'-O-met	Q5A0V9	NOP1	CAALFM_C406720WA	1.823	1.108	0.075
Ayr2p	A0A1D8PQ42	AYR2	CAALFM_C603270CA	1.823	1.108	0.075
Phosphatidylinositol transfer protein SFH5 (PITP SFH5	Q5AP66	SFH5	CAALFM_C110270CA	1.823	1.108	0.075
Uso6p	A0A1D8PK69	USO6	CAALFM_C305310WA	1.823	1.108	0.075
Nucleolar protein 58	Q59S06	NOP58	CAALFM_C600370CA	1.823	1.108	0.075
1-phosphatidylinositol 4-kinase	A0A1D8PTE0	STT4	CAALFM_CR07090WA	1.823	1.108	0.075
Mitochondrial presequence protease (EC 3.4.24.-)	Q5A301	CYM1	CAALFM_C306230WA	1.823	1.108	0.075
Uncharacterized protein	A0A1D8PPV6	orf19.3475	CAALFM_C602330WA	10.024	5.540	0.112
Glutathione reductase (EC 1.8.1.7)	Q59NQ5	GLR1	CAALFM_C501520CA	10.024	5.540	0.112
Uncharacterized protein	A0A1D8PDL7	orf19.2489	CAALFM_C105630CA	10.024	5.540	0.112
Peroxisomal catalase (EC 1.11.1.6)	O13289	CAT1	CAALFM_C106810WA	16.402	8.863	0.121
Protein phosphatase 2A structural subunit	Q5ADN1	TPD3	CAALFM_C306910CA	8.201	4.432	0.121
Ist2p	A0A1D8PE41	IST2	CAALFM_C107520CA	14.580	7.755	0.127
Ribosomal 40S subunit protein S14B	A0A1D8PDT3	RPS14B	CAALFM_C106450CA	6.379	3.324	0.135
Ribosomal 40S subunit protein S20	Q5A389	RPS20	CAALFM_CR08150WA	10.935	5.540	0.146
ATP-dependent 6-phosphofructokinase (ATP-PFK) (Ph	Q5AK53	PFK1	CAALFM_C504810WA	10.935	5.540	0.146
UTP--glucose-1-phosphate uridylyltransferase (EC 2.7	Q59KI0	UGP1	CAALFM_CR04660CA	15.491	7.755	0.151
Zn(2+) transporter	A0A1D8PGH6	orf19.1534	CAALFM_C202180WA	9.112	4.432	0.162
Uncharacterized protein	A0A1D8PNE5	orf19.4246	CAALFM_C502380WA	9.112	4.432	0.162
Ribosomal 40S subunit protein S3	A0A1D8PSV5	RPS3	CAALFM_CR04810WA	9.112	4.432	0.162
40S ribosomal protein S21	A0A1D8PCG7	RPS21B	CAALFM_C101370CA	4.556	2.216	0.162
Ribosomal 60S subunit protein L31B	A0A1D8PHF5	orf19.3572.3	CAALFM_C205410WA	4.556	2.216	0.162
Ribosomal 40S subunit protein S13	A0A1D8PPE0	RPS13	CAALFM_C600650CA	4.556	2.216	0.162
Dipeptidyl peptidase 3 (EC 3.4.14.4) (Dipeptidyl amin	A0A1D8PQB4	orf19.5773	CAALFM_C603960WA	4.556	2.216	0.162

3-hydroxyisobutyryl-CoA hydrolase, mitochondrial (E	Q5AI24	EHD3	CAALFM_C103320CA	4.556	2.216	0.162
Glyco_hydro_63 domain-containing protein	Q5AED0	orf19.338	CAALFM_C303410CA	22.781	11.079	0.162
Citrulline--aspartate ligase (EC 6.3.4.5)	A0A1D8PRR5	ARG1	CAALFM_CR00620CA	13.669	6.648	0.162
Acyl-coenzyme A oxidase	Q5AJD9	POX1-3	CAALFM_C301960CA	20.959	9.971	0.170
Ribosomal 40S subunit protein S18B	A0A1D8PQQ5	RPS18	CAALFM_C700960WA	11.846	5.540	0.177
Alpha-mannosidase	Q5AF38	AMS1	CAALFM_C402360WA	41.006	18.835	0.184
Ribosomal 40S subunit protein S9B	A0A1D8PGY8	RPS9B	CAALFM_C203820CA	7.290	3.324	0.187
Ribosomal 40S subunit protein S11A	A0A1D8PN83	orf19.4149.1	CAALFM_C501540WA	7.290	3.324	0.187
Carbamoyl-phosphate synthase (Glutamine-hydrolyz	Q5A8A6	CPA2	CAALFM_CR01330WA	7.290	3.324	0.187
Protein FMP52, mitochondrial	Q5AP65	FMP52	CAALFM_C110280CA	7.290	3.324	0.187
60S ribosomal protein L10a	Q9UVJ4	RPL10A	CAALFM_C602240CA	7.290	3.324	0.187
Methylmalonate-semialdehyde dehydrogenase (CoA	A0A1D8PM94	ALD6	CAALFM_C405130CA	10.024	4.432	0.199
Cip1p	A0A1D8PPI6	CIP1	CAALFM_C601070CA	12.757	5.540	0.206
40S ribosomal protein S1 (S3aE)	P40910	RPS1	CAALFM_C103090WA	12.757	5.540	0.206
H(+)-transporting V1 sector ATPase subunit E	A0A1D8PS38	VMA4	CAALFM_CR01970CA	15.491	6.648	0.211
Ribosomal 60S subunit protein L7A	A0A1D8PDL6	orf19.2478.1	CAALFM_C105720WA	10.935	4.432	0.233
Histone H4	Q59VN4	HHF1	CAALFM_C104240CA	10.935	4.432	0.233
40S ribosomal protein S6	A0A1D8PL99	RPS6A	CAALFM_C401270WA	10.935	4.432	0.233
60S ribosomal protein L36	A0A1D8PH21	RPL39	CAALFM_C203960WA	2.734	1.108	0.233
RuvB-like helicase 1 (EC 3.6.4.12)	Q5A0W7	RVB1	CAALFM_C406800WA	2.734	1.108	0.233
Glucan 1,3-beta-glucosidase 2 (EC 3.2.1.58) (Exo-1,3-b	Q5AIA1	EXG2	CAALFM_C102630CA	2.734	1.108	0.233
E2 ubiquitin-conjugating protein	Q5A513	orf19.933	CAALFM_C500560WA	2.734	1.108	0.233
Nuc2p	Q5AH07	NUC2	CAALFM_C701900WA	2.734	1.108	0.233
Ribosomal 40S subunit protein S10A	A0A1D8PI15	RPS10	CAALFM_C208040CA	2.734	1.108	0.233
RNA export factor	A0A1D8PQE5	GLE2	CAALFM_C604360CA	2.734	1.108	0.233
V-SNARE protein	A0A1D8PJW1	VTI1	CAALFM_C303400CA	2.734	1.108	0.233
Covalently-linked cell wall protein 14	Q5AFN8	SSR1	CAALFM_C700860WA	2.734	1.108	0.233
Proteasome regulatory particle lid subunit	A0A1D8PD15	RPN3	CAALFM_C103520WA	2.734	1.108	0.233
V-type proton ATPase subunit C	Q5A2U9	VMA5	CAALFM_C208190WA	2.734	1.108	0.233
Ribosomal 40S subunit protein S17B	A0A1D8PEY9	RPS17B	CAALFM_C110870WA	8.201	3.324	0.233
Glutamine--fructose-6-phosphate aminotransferase [	P53704	GFA1	CAALFM_C302280CA	8.201	3.324	0.233
Vacuolar protein 8	Q59MN0	VAC8	CAALFM_C405150WA	8.201	3.324	0.233
Sphingolipid C9-methyltransferase (C-9-MT) (EC 2.1.1	Q5APD4	MTS1	CAALFM_C109680WA	8.201	3.324	0.233
40S ribosomal protein S7	Q5AJ93	RPS7A	CAALFM_C301490WA	8.201	3.324	0.233
V-type proton ATPase subunit H	A0A1D8PSX0	VMA13	CAALFM_CR05080WA	5.468	2.216	0.233
60S ribosomal protein L13	O59931	RPL13	CAALFM_C103020CA	5.468	2.216	0.233
Fumarate hydratase (EC 4.2.1.2)	Q5A6L1	FUM11	CAALFM_CR04530WA	5.468	2.216	0.233
Trehalose-phosphatase	Q5AI14	TPS2	CAALFM_C103380WA	5.468	2.216	0.233
Gvp36p	Q5A473	GVP36	CAALFM_C405550CA	5.468	2.216	0.233
Ribosomal 60S subunit protein L28	A0A1D8PSC5	RPL28	CAALFM_CR03030CA	5.468	2.216	0.233
Vacuolar transporter chaperone	A0A1D8PSJ6	VTC3	CAALFM_CR03610CA	5.468	2.216	0.233
Thioredoxin peroxidase	Q5A5A0	PRX1	CAALFM_C702810WA	5.468	2.216	0.233
Cysteine proteinase 1, mitochondrial (EC 3.4.22.40)	Q5A6L5	LAP3	CAALFM_CR04480CA	17.314	6.648	0.254
Ribosomal 60S subunit protein L4B	A0A1D8PFV1	RPL4B	CAALFM_C114110CA	14.580	5.540	0.258
60S ribosomal protein L6	A0A1D8PCX8	RPL6	CAALFM_C103110WA	11.846	4.432	0.264
Ubiquinol--cytochrome-c reductase catalytic subunit	A0A1D8PHA3	CYT1	CAALFM_C204950CA	12.757	4.432	0.293
4-alpha-glucanotransferase (EC 2.4.1.25) (EC 3.2.1.33	Q59MN2	GDB1	CAALFM_C405140CA	6.379	2.216	0.293
Ribosomal 60S subunit protein L16A	Q5AB87	RPL16A	CAALFM_C100180WA	6.379	2.216	0.293
Uncharacterized protein	A0A1D8PCX0	orf19.3003	CAALFM_C103100WA	14.580	4.432	0.346
Uncharacterized protein	A0A1D8PTW2	orf19.7310	CAALFM_CR09140CA	18.225	5.540	0.346
Tricalbin	A0A1D8PEY6	orf19.1840	CAALFM_C110680CA	7.290	2.216	0.346
Protein transport protein SSO2	Q59YF0	SSO2	CAALFM_C209740WA	7.290	2.216	0.346
Cytochrome c peroxidase, mitochondrial (CCP) (EC 1.	Q5AEN1	CCP1	CAALFM_C302480CA	7.290	2.216	0.346
Arf3p	A0A1D8PJ64	ARF3	CAALFM_C301470WA	3.645	1.108	0.346
Ribosomal 60S subunit protein L32	A0A1D8PPN6	RPL32	CAALFM_C601700WA	3.645	1.108	0.346

Calcium channel YVC1 (Vacuolar transiant receptor p	Q5A2J7	YVC1	CAALFM_C207730WA	3.645	1.108	0.346
Uncharacterized protein	A0A1D8PF63	orf19.1152	CAALFM_C111670WA	3.645	1.108	0.346
Sterol 24-C-methyltransferase (EC 2.1.1.41) (Delta(24	O74198	ERG6	CAALFM_C302150CA	3.645	1.108	0.346
Emp24p	Q59V63	EMP24	CAALFM_CR07590WA	3.645	1.108	0.346
Ribosomal 60S subunit protein L23B	A0A1D8PPT5	RPL23A	CAALFM_C602070CA	3.645	1.108	0.346
AA_permease domain-containing protein	A0A1D8PQM7	orf19.7056	CAALFM_C700630CA	3.645	1.108	0.346
Actin-related protein 2/3 complex subunit	A0A1D8PT60	ARC40	CAALFM_CR06180WA	3.645	1.108	0.346
E3 ubiquitin ligase complex SCF subunit	Q59WE2	SKP1	CAALFM_C107410CA	3.645	1.108	0.346
Induced during hyphae development protein 1 (GPI-a	Q5A8I8	IHD1	CAALFM_C603850CA	3.645	1.108	0.346
Exportin	A0A1D8PRR9	CRM1	CAALFM_CR00520CA	3.645	1.108	0.346
Histone H3.1/H3.2	Q59VN2	HHT21	CAALFM_C104260WA	3.645	1.108	0.346
Histone H2B.1	P48989	HTB1	CAALFM_C303900CA	15.491	4.432	0.369
Low-affinity Zn(2+) transporter	A0A1D8PGN5	ZRT2	CAALFM_C202590WA	12.757	3.324	0.406
Ribosomal 40S subunit protein S2	Q5A900	RPS21	CAALFM_C101480CA	9.112	2.216	0.433
ATP-binding cassette family ATPase	Q5A2T2	KRE30	CAALFM_C208000CA	4.556	1.108	0.433
Ribosomal protein L19	A0A1D8PK40	RPL19A	CAALFM_C304500CA	4.556	1.108	0.433
Hsp30p	Q59TA5	HSP30	CAALFM_C101990WA	4.556	1.108	0.433
Ribosomal 60S subunit protein L33A	A0A1D8PHH4	orf19.6882.1	CAALFM_C205710CA	4.556	1.108	0.433
Zrt1p	A0A1D8PMR6	ZRT1	CAALFM_C406970CA	10.024	2.216	0.470
Histone H2A.2	Q59VP2	HTA2	CAALFM_C104170CA	10.935	2.216	0.504
60S ribosomal protein L27	A0A1D8PFG4	RPL27A	CAALFM_C112390CA	5.468	1.108	0.504
Sla2p	A0A1D8PRF5	SLA2	CAALFM_C703810WA	5.468	1.108	0.504
60S ribosomal protein L8	A0A1D8PF11	RPL82	CAALFM_C111030WA	11.846	2.216	0.536
Pyr_redox_2 domain-containing protein	A0A1D8PI22	orf19.2175	CAALFM_C208100WA	6.379	1.108	0.565
Ribosomal 60S subunit protein L10	Q5AIB8	RPL10	CAALFM_C102460WA	6.379	1.108	0.565
Ribosomal 60S subunit protein L26B	A0A1D8PCQ5	orf19.3690.2	CAALFM_C102330CA	7.290	1.108	0.617
Uncharacterized protein	A0A1D8PRQ2	orf19.7502	CAALFM_CR00310CA	7.290	1.108	0.617
Ribosomal 40S subunit protein S19A	A0A1D8PK61	RPS19A	CAALFM_C305200WA	8.201	1.108	0.663
Ribosomal 60S subunit protein L14B	A0A1D8PFL9	RPL14	CAALFM_C113050WA	9.112	1.108	0.704
60S ribosomal protein L20	A0A1D8PLC9	RPL20B	CAALFM_C401520CA	9.112	1.108	0.704
Ribosomal 60S subunit protein L18A	A0A1D8PK43	RPL18	CAALFM_C305100CA	10.024	1.108	0.742
**Proteins inhibited by DiMIQ**						
Aldo-keto reductase superfamily protein	Q5ADM5	orf19.6816	CAALFM_C306860CA	10.024	0.000	drug inhibited
Glutamate synthase (NADH)	A0A1D8PDU9	GLT1	CAALFM_C106550WA	8.201	0.000	drug inhibited
Flavohemoprotein (EC 1.14.12.17) (Flavohemoglobin)	Q59MV9	YHB1	CAALFM_CR07790CA	7.290	0.000	drug inhibited
Vtc4p	Q59PH5	VTC4	CAALFM_C403360CA	6.379	0.000	drug inhibited
40S ribosomal protein S22-B	P0CU35	RPS22B	CAALFM_C103620CA	5.468	0.000	drug inhibited
4-aminobutyrate aminotransferase (EC 2.6.1.19) (GABA	A0A1D8PH55	UGA1	CAALFM_C204190CA	4.556	0.000	drug inhibited
Histone H1	A0A1D8PR93	HHO1	CAALFM_C703180CA	3.645	0.000	drug inhibited
Acetyl-CoA C-acyltransferase	A0A1D8PRL6	POT1	CAALFM_CR00150CA	3.645	0.000	drug inhibited
Uncharacterized protein	Q5A4L1	orf19.3430	CAALFM_C601560WA	2.734	0.000	drug inhibited
V-type proton ATPase subunit D (V-ATPase subunit D	P87220	VMA8	CAALFM_C406400CA	2.734	0.000	drug inhibited
Ribosomal 60S subunit protein L17B	Q59TE0	RPL17B	CAALFM_C204600CA	2.734	0.000	drug inhibited
Ribosomal 40S subunit protein S23B	A0A1D8PDU3	RPS23A	CAALFM_C106580WA	2.734	0.000	drug inhibited
Asr2p	A0A1D8PTU7	ASR2	CAALFM_CR08890CA	2.734	0.000	drug inhibited
Histone H2A.Z	Q5AEE1	HTZ1	CAALFM_C303280CA	2.734	0.000	drug inhibited
Glutamine synthetase (EC 6.3.1.2)	A0A1D8PSY1	GLN1	CAALFM_CR05050WA	3.645	0.000	drug inhibited
Virulence protein SSD1	Q5AK62	SSD1	CAALFM_C504730CA	2.734	0.000	drug inhibited
Ribosomal 60S subunit protein L43A	A0A1D8PP14	RPL43A	CAALFM_C504590CA	2.734	0.000	drug inhibited
Ahp2p	A0A1D8PR39	AHP2	CAALFM_C702390WA	2.734	0.000	drug inhibited
Actin-related protein 3	Q59Z11	ARP3	CAALFM_C207320WA	2.734	0.000	drug inhibited
Cta3p	A0A1D8PF53	CTA3	CAALFM_C111540CA	2.734	0.000	drug inhibited
pH-responsive protein 2 (pH-regulated protein 2)	O13318	PHR2	CAALFM_C100220WA	1.823	0.000	drug inhibited
Methylglyoxal reductase (NADPH-dependent)	Q5ABT9	orf19.5611	CAALFM_C603240WA	1.823	0.000	drug inhibited
Putative cystathionine beta-lyase	A0A1D8PTV7	orf19.7297	CAALFM_CR09010CA	1.823	0.000	drug inhibited

Trehalose 6-phosphate synthase/phosphatase compl	A0A1D8PIS4	TPS3	CAALFM_C210690WA	2.734	0.000	drug inhibited
Uncharacterized protein	Q5ALU6	orf19.1480	CAALFM_C201690WA	1.823	0.000	drug inhibited
Ribosomal 40S subunit protein S28B	A0A1D8PQN0	RPS28B	CAALFM_C700710WA	1.823	0.000	drug inhibited
Rab family GTPase	Q59X89	VPS21	CAALFM_CR08060CA	1.823	0.000	drug inhibited
Acid phosphatase (EC 3.1.3.2)	A0A1D8PE91	LTP1	CAALFM_C108260CA	1.823	0.000	drug inhibited
Peroxisome biogenesis protein	Q5ACV4	orf19.2113	CAALFM_C200190CA	1.823	0.000	drug inhibited
Uncharacterized protein	Q5ABD5	orf19.6035	CAALFM_C100700WA	1.823	0.000	drug inhibited
ATP-dependent RNA helicase DBP5 (EC 3.6.4.13)	Q5AJD0	DBP5	CAALFM_C301860CA	1.823	0.000	drug inhibited
Uncharacterized protein	A0A1D8PP33	orf19.3983	CAALFM_C504950CA	1.823	0.000	drug inhibited
Siderophore transporter	Q5A2T6	SIT1	CAALFM_C208050CA	1.823	0.000	drug inhibited
Ccc1p	Q59SS1	CCC1	CAALFM_C303710WA	1.823	0.000	drug inhibited
Phosphatidylinositol 4,5-bisphosphate-binding prote	A0A1D8PPT0	SLM2	CAALFM_C602060WA	1.823	0.000	drug inhibited
ERF-3 (ERF2) (Eukaryotic peptide chain release factor	Q59YE8	SUP35	CAALFM_C209720WA	1.823	0.000	drug inhibited
Importin-alpha export receptor	A0A1D8PE78	orf19.1229	CAALFM_C107690CA	1.823	0.000	drug inhibited
Golgi apparatus membrane protein TVP18	Q5APC0	TVP18	CAALFM_C109800CA	1.823	0.000	drug inhibited
Ribosomal protein L37	A0A1D8PF45	RPL37B	CAALFM_C111360WA	1.823	0.000	drug inhibited
Ribosomal 40S subunit protein S29A	A0A1D8PTR4	orf19.6415.1	CAALFM_CR08480CA	1.823	0.000	drug inhibited
Regulator of cytoskeleton and endocytosis RVS167	Q59LF3	RVS167	CAALFM_C604040CA	1.823	0.000	drug inhibited
DNA repair protein	Q5AGX1	SMC6	CAALFM_C701580WA	1.823	0.000	drug inhibited
IFRD domain-containing protein	A0A1D8PDV1	orf19.6245	CAALFM_C106660WA	1.823	0.000	drug inhibited
Ecm29p	A0A1D8PKU7	ECM29	CAALFM_C307260CA	1.823	0.000	drug inhibited
Uncharacterized protein	A0A1D8PKK4	orf19.6832	CAALFM_C306710WA	1.823	0.000	drug inhibited
Serine/threonine-protein kinase GIN4 (EC 2.7.11.1) (G	Q59W62	GIN4	CAALFM_C111400CA	1.823	0.000	drug inhibited
Phosphatidylinositol-3-phosphate-binding protein	A0A1D8PLB6	BEM1	CAALFM_C401410WA	1.823	0.000	drug inhibited
Glucosamine-6-phosphate isomerase (EC 3.5.99.6) (G	Q04802	NAG1	CAALFM_C604590CA	1.823	0.000	drug inhibited
Hsp70 nucleotide exchange factor FES1	Q59NN8	FES1	CAALFM_C600760WA	1.823	0.000	drug inhibited
Uncharacterized protein	Q5A7P8	orf19.5418	CAALFM_C300470WA	1.823	0.000	drug inhibited
Glutamate dehydrogenase	A0A1D8PMH8	GDH3	CAALFM_C406120WA	1.823	0.000	drug inhibited
SUMO ligase	A0A1D8PCK0	SIZ1	CAALFM_C101560WA	1.823	0.000	drug inhibited
CTP synthase (EC 6.3.4.2) (UTP--ammonia ligase)	Q5AK79	URA7	CAALFM_C504570CA	1.823	0.000	drug inhibited
Inheritance of peroxisomes protein 1	Q5AH13	orf19.6525	CAALFM_C701960WA	1.823	0.000	drug inhibited
Uncharacterized protein	Q5A925	orf19.3309	CAALFM_C101210WA	1.823	0.000	drug inhibited
PH domain-containing protein	A0A1D8PMG9	orf19.4715	CAALFM_C406130WA	1.823	0.000	drug inhibited
Uncharacterized protein	A0A1D8PQS3	orf19.7006	CAALFM_C701070CA	1.823	0.000	drug inhibited
Prn4p	Q5AA23	PRN4	CAALFM_C105880WA	1.823	0.000	drug inhibited
SAGA complex subunit	A0A1D8PNJ5	orf19.4312	CAALFM_C502900WA	1.823	0.000	drug inhibited
Uncharacterized protein	A0A1D8PMC0	orf19.1240	CAALFM_C405590WA	1.823	0.000	drug inhibited
Uncharacterized protein	A0A1D8PG45	orf19.2070	CAALFM_C200570WA	2.734	0.000	drug inhibited
Uncharacterized protein	Q5ADQ2	orf19.6789	CAALFM_C307110WA	1.823	0.000	drug inhibited
